# Corticostriatal cocaine-seeking ensembles are defined by differing gene expression from sucrose-seeking ensembles using a within-subject dual self-administration and seeking mouse model

**DOI:** 10.1016/j.addicn.2025.100242

**Published:** 2025-11-21

**Authors:** Carl G. Litif, Levi T. Flom, Kathryn L. Sandum, Skylar L. Hodgins, Lucio Vaccaro, Jerry A. Stitzel, Nathan Ungerleider, Maria Constanza Mannino, Jason P. Gigley, Todd A. Schoborg, Ana-Clara Bobadilla

**Affiliations:** a School of Pharmacy, University of Wyoming, Laramie, WY, USA; b Department of Integrative Physiology, University of Colorado Boulder, Boulder, CO, USA; c Institute for Behavioral Genetics, University of Colorado Boulder, Boulder, CO, USA; d Department of Molecular Biology, University of Wyoming, Laramie, WY, USA; e Department of Biomedical Sciences, Colorado State University, Fort Collins, CO, USA; f Department of Molecular Microbiology and Immunology, Saint Louis University School of Medicine, St. Louis, MO, USA; g Department of Psychiatry, University of California San Diego School of Medicine, La Jolla, CA, USA; h Instituto de Investigaciones Bioquímicas de La Plata, Facultad de Ciencias Médicas (UNLP) La Plata, Argentina

**Keywords:** Cocaine, Sucrose, Neuronal ensembles, RNAsequencing

## Abstract

Recurrent cocaine seeking is a hallmark of cocaine use disorder. To develop therapeutic targets, it is critical to understand the neurobiological changes specific to cocaine-seeking in context with the seeking of non-drug rewards, e.g., sucrose. The nucleus accumbens (NAc) and medial prefrontal cortex (mPFC) are known regions associated with cocaineand sucrose-seeking ensembles, i.e., a sparse population of co-activated neurons linked with behavior. Within ensembles, transcriptomic alterations in the NAc and mPFC underlie the learning and recall of cocaine- and sucrose-seeking behavior. However, the transcriptomics exclusively driving cocaine seeking independent from sucrose seeking have not yet been defined using a within-subject approach. Using Ai14:cFos-TRAP2 transgenic mice in a dual cocaine and sucrose self-administration model, we fluorescently sorted and characterized the transcriptomes defining cocaine-seeking in reference to the sucrose-seeking ensemble, overlapping ensemble in between cocaine and sucrose-seeking, and the non-ensemble population. Our data suggests there are robust transcriptomic changes linked with cocaine-seeking that differ from sucrose-seeking ensembles and the non-ensemble population which could guide future studies aimed to detangle cocaine-seeking behavior without altering non-drug reward seeking.

## Introduction

Cocaine use disorder (CUD) is characterized by the recurrent seeking of cocaine after periods of abstinence, often leading to relapse [1]. The absence of pharmaceutical intervention for attenuating cocaine seeking is partially due to a lack of understanding of the discrete transcriptomic alterations driving the rewarding effects of cocaine apart from non-drug rewards, e.g., sucrose. It is known that cocaine and sucrose seeking both involve the recruitment of sparse activity-dependent neural circuits, defined as neuronal ensembles [2], within the nucleus accumbens (NAc) and medial prefrontal cortex (mPFC) [3–6]. Recently it was shown ensembles linked to cue-associated cocaine and sucrose seeking are mostly separate despite having an overlapping population of neurons [7–8]. Mounting evidence also suggests contingent reward intake facilitates long-lasting alterations in gene expression within the NAc and mPFC to drive reward-seeking behavior [9–14]. However, studies focused on transcriptomic expression in cocaine or sucrose seeking often lack within-subject comparisons between drug and non-drug rewards.

Considering previous findings characterizing the relationship between drug and non-drug rewards, we hypothesized neuronal ensembles linked with cocaine- and sucrose-seeking have differing yet overlapping transcriptomic landscapes. To test this hypothesis, we used a within-subject approach to characterize sex-considerate and region-dependent gene expression profiles for cocaine and sucrose seeking in mice. Using mice featuring the targeted recombination in active populations (TRAP2) dual-event tagging scheme [15] and fluorescent activated cell sorting [16,10], we identified and isolated ensembles in the NAc and mPFC from two temporally separate cocaine- and sucrose-seeking events. We then used bulk RNA-sequencing (RNAseq) to compare significant sex-, region-, and reward-specific transcriptomic profiles for cocaine-seeking in comparison to multiple references (sucrose-seeking ensemble, overlapping cocaine- and sucrose-seeking ensemble, or non-ensemble population) to gain comprehensive insight into the transcriptomic mechanisms likely related only to cocaine-seeking behavior. Using this approach, we developed a repository of sex-considerate transcriptomic information to define distinct or intersecting alterations in gene expression for cocaine- and sucrose-seeking ensembles in the NAc and mPFC.

## Results

### Cue-induced seeking of cocaine and sucrose recruit reward-specific ensembles

**Fig. 1.**
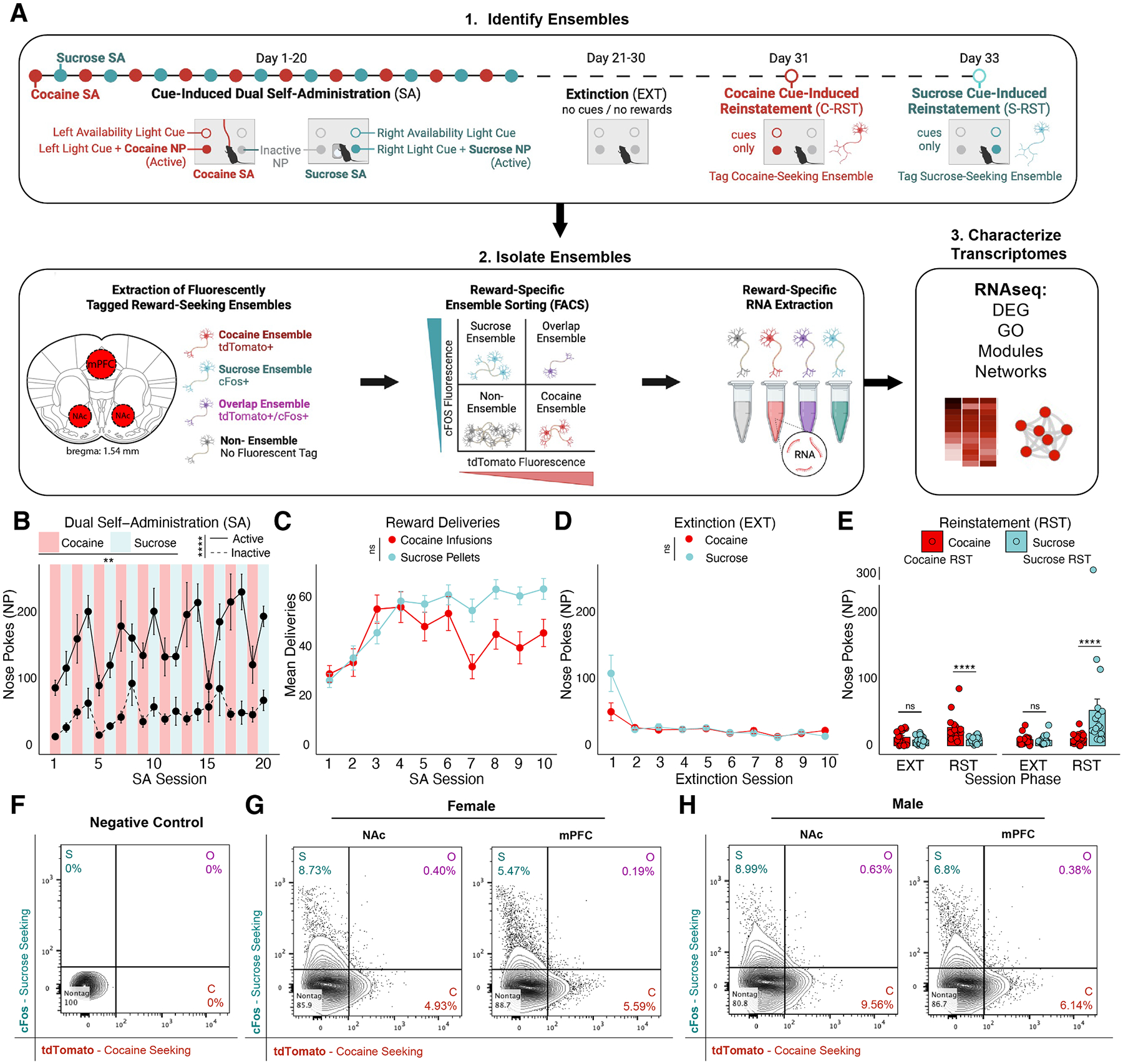
Behavioral paradigm, cell sorting, and ensemble tagging strategy for transcriptomic profiling of reward-seeking neurons. **A** Schematic of the experimental workflow. Mice underwent dual self-administration (SA) of cue-paired cocaine and sucrose across alternating days (Day 1–20), followed by extinction sessions without rewards or cues (Day 21–30, 32), and cue-induced reinstatement (RST) sessions (Day 31 and 33) for cocaine (C-RST) or sucrose (S-RST) seeking. Cocaine-seeking ensembles were tagged via TRAP2-tdTomato labeling during C-RST while sucrose-seeking ensembles were identified by Fos expression during S-RST. Fluorescently labeled neurons from NAc and mPFC were isolated via fluorescence activated cell sorting (FACS) and processed for RNA sequencing. **B** Active and inactive nose poke (NP) responses across 20 dual SA sessions. Red and blue background bars indicate cocaine and sucrose SA days, respectively. GLMM: **p 〈 0.01, ****p < 0.0001. **C** Cocaine infusions and sucrose pellet deliveries during their respective SA sessions. **D** Cocaine- or sucrose-trained active NP behavior during extinction sessions. GLMM: ns p 〉 0.05. **E** Cue-induced reinstatement of cocaine (red) or sucrose (blue) seeking behavior, compared to the first 30 min of the previous extinction day (EXT). GLMM: ns *p* > 0.05, ****p < 0.0001. **F** Negative control FACS plot from a non-induced control animal, showing minimal fluorescence in all quadrants. **G,H** Representative FACS quadrant plots from female (**G**) and male (**H**) NAc and mPFC. Neuronal populations were gated as: cocaine-seeking (C; bottom-right, tdTomato^+^/cFos^−^), sucrose-seeking (S; top-left, cFos^+^/tdTomato^−^), overlapping ensemble (O; top-right, tdTomato^+^/cFos^+^), and non-ensemble (Nontag; bottom-left, tdTomato^−^/cFos^−^). Percentages indicate the proportion of total NeuN^+^ events from 1000,000 recorded. See Supplementary Figure 3 for full replicate gating strategy.

**Fig. 2.**
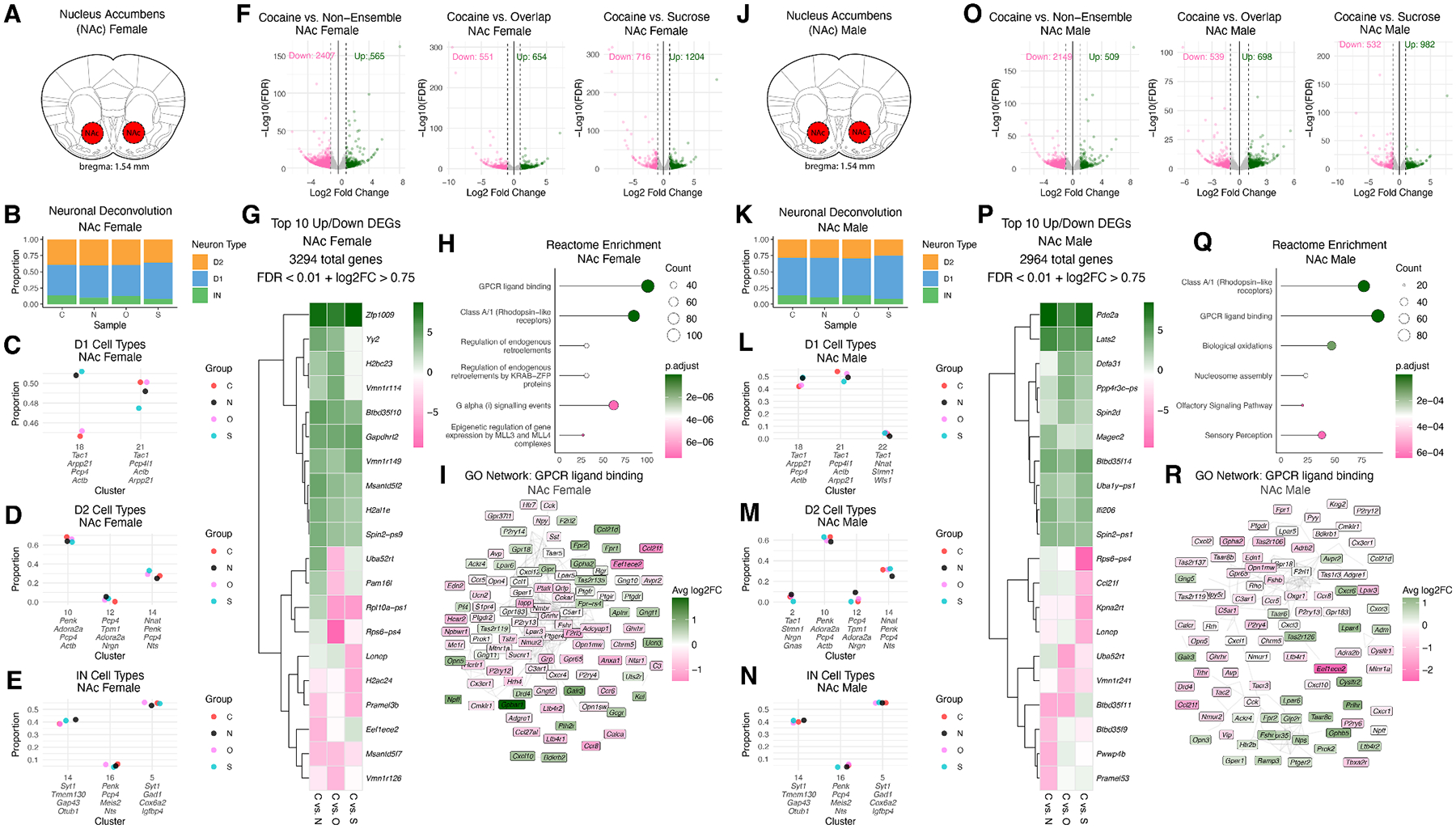
Transcriptomic of cocaine-seeking ensembles in the nucleus accumbens (NAc). **A,J** Atlas schematic showing the NAc dissection site used for bulk RNA-seq in female (**A**) and male mice (**J**; mice per sex: *n* = 9 mice grouped into 3 replicates of 3-pooled mice; see Supplemental Figure 1 for full experimental workflow). **B, K** MuSiC-based deconvolution of bulk RNA-seq showing estimated proportions of D1- (D1) or D2-like medium spiny neurons (D2), and interneurons (IN) across ensemble-defined groups: cocaine-seeking ensemble (C), non-ensemble (N), overlapping ensemble (O), and sucrose-seeking ensemble (S) - in female (**B**) and male (**K**) NAc. **C,E,L,N** Refined deconvolution results for D1 (**C, L**), D2 (**D, M**), and IN (**E, N**) neuronal subclusters in female (**C–E**) and male (**L–N**) NAc. Each point reflects estimated proportions per cluster across groups; cluster labels include top four marker genes by log_2_ fold change. **F,O** Volcano plots showing differentially expressed genes (DEGs) after comparing the cocaine-seeking ensemble with the non-ensemble, sucrose-seeking, or overlapping ensemble as the reference in female (**F**) and male (**O**) NAc. Genes meeting FDR < 0.01 and the absolute value (abs) of log_2_FC > 0.75 criteria are colored (pink: downregulated, green: upregulated) while the genes that did not meet the criteria are grey. **G,P** Heatmaps showing the top 10 upregulated and downregulated DEGs per contrast in female (**G**) and male (**P**) NAc, based on log_2_ fold change. **H,Q** Dot plots showing Reactome pathway enrichment of DEGs combined from all of the criteria-met DEG from each comparison in female (**H**) and male (**Q**) NAc. Dot size reflects the number of DEGs per pathway; color indicates adjusted p-value. **I,R** GO network diagrams highlighting the top enriched Reactome pathway in female (**I**) and male (**R**) NAc. Genes are colored by average log_2_ fold change (dark green = upregulated; hot pink = downregulated).

To characterize cocaine-seeking ensembles in the context of sucrose-seeking, we used a dual self-administration (SA), extinction (EXT), and cocaine- or sucrose-specific cue-induced reinstatement behavioral model in Ai14:cFos-TRAP2 mice to first label both cocaine- and sucrose-seeking ensembles. Behavior was followed by fluorescent activated cell sorting (FACS) and downstream bulk RNA sequencing (RNAseq) to characterize transcriptomic profiles in fluorescently labeled cocaine- and sucrose-seeking ensembles (Fig. 1a, Supplemental Figure 1A). Mice (*n* = 9 female, 9 male) underwent alternating daily sessions of intravenous cocaine and oral sucrose SA on a Fixed Ratio 1 (FR1) schedule (Fig. 1b) for 20 days (10 sessions per reward). SA was contingent on cue-paired interaction with cocaine- or sucrose-reinforced active nose pokes (NP) in counterpart to an additional non-reinforced inactive NP without consequences. Cocaine and sucrose SA sessions showed consistent discrimination between the active NP and the inactive NP for both reward types (Nose Poke (Active vs. Inactive), estimate = 1.574, SE = 0.258, *p* < 0.0001; Supplemental Table 1a). A significant interaction between reward type and sex was observed (Reward x Sex: estimate = −0.902, SE = 0.386, *p* = 0.0194; Supplemental Table 1a), indicating that the effect of reward on nose-poke discrimination differed by sex. Post-hoc comparisons revealed that female mice exhibited greater discrimination for cocaine than sucrose, while male mice showed the opposite pattern, with stronger discrimination for sucrose than cocaine (Supplemental Figure 2A, Supplemental Table 1a). Reward deliveries showed a significant interaction between reward type and session (Reward x Session: estimate = 0.073, SE = 0.026, *p* = 0.0061; Fig. 2c; Supplemental Figure 2B, Supplemental Table 1b).

Following SA, mice underwent EXT in the original SA chamber without reward administration or cue exposure. During EXT, mice decreased interaction of NPs previously paired with cocaine or sucrose during SA over the course of the 10 days (Nose Poke (Sucrose vs. Cocaine): estimate = 0.387, SE = 0.198, *p* = 0.0505; Fig. 1d, Supplemental Table 1c; Supplemental Figure 2C, Supplemental Table 1c). Comparing the overall reward-paired active nose-poke interactions between the SA and EXT phases showed a significant reduction of pressing during the EXT phase (Active Nose Poke (SA vs. EXT): estimate = −1.774, SE = 0.094, *p* < 0.0001; SupplementalTable 1d).

To induce seeking of cocaine or sucrose, mice underwent cocaine(C-RST) and sucrose-specific (S-RST) cued-reinstatement sessions where mice were exposed to reward-specific cues in the absence of reward administration. Mice displayed cocaine seeking during the C-RST session, as demonstrated by increased interaction with the cocaine-paired active NP compared to the inactive NP (i.e., sucrose-paired NP) and to the cocaine-paired active NP during the EXT session from the previous day (Fig. 1e) with no apparent sex differences (C-RST × Nose Poke: estimate = −0.968, SE = 0.380, *p* = 0.0108; Supplemental Figure 2D; Supplemental Table 1e). Immediately following C-RST, 4-hydroxytamoxifen (4-OHT) was administered to permanently tag active cFos-expressing neurons considered the cocaine-seeking ensemble with fluorescent tdTomato. In parallel, mice displayed sucrose seeking during the S-RST session, as observed by increased interaction with the sucrose-paired active NP compared to the inactive NP and the sucrose-paired active NP during the previous EXT session (Fig. 1e) again with no apparent sex differences (S-RST × Nose Poke: estimate = 1.486, SE = 0.369, *p* = <0.0001; Supplemental Figure 2D; Supplemental Table 1e). Mice were euthanized 60 min post-S-RST to capture endogenous cFos protein concentration using immunocytochemistry (ICC) for neurons comprising what is considered the sucrose-seeking ensemble, while the tagged cocaine-seeking ensemble remains an expression of the tdTomato tag.

Immediately following euthanasia, we dissociated neurons from the NAc (primarily lateral accumbens shell and lateral accumbens core) and mPFC (primarily prelimbic and cingulate areas 1 and 2) regions and used FACS to separate neuronal cocaine-seeking ensemble, sucrose-seeking ensemble, overlapped ensemble, and non-ensemble populations. After debris removal (Supplemental Figure 3A), we used an Ai14:cFos-TRAP2 control mouse with only a neuronal marker (NeuN+, Supplemental Figure 3B) and no induced ensemble tagging (Supplemental Figure 3C) to identify and sort the cocaine-seeking ensemble (C; lower right quadrant; tdTomato+), sucrose-seeking ensemble (S; upper left quadrant; cFos+), overlapping ensemble (O; upper right quadrant; tdTomato+/cFos+), and the non-ensemble (bottom left quadrant) for females (Supplementary Figure 3D–G, and males (Supplementary Figure 3H–K). Additional flow cytometry analysis showed a significant interaction between the size of the ensemble based condition and region (Condition × Region: estimate = 67.66, SE = 33.83, *p* = 0.0232; Supplemental Figure 3L; Supplemental Table 1f). After identifying and isolating cocaine-seeking, sucrose-seeking, and overlapping ensembles (Fig. 1f–h), we proceeded with transcriptomic characterization.

### Cocaine and sucrose seeking ensembles are region- and sex-dependent

We sequenced the RNA of lysates from reward-specific seeking ensembles (Supplemental Figure 1) to bioinformatically detect significant transcriptomic patterns related to cocaine-seeking. Raw count data of all samples were normalized and filtered to remove low-count background noise followed by principal component analysis (PCA). The PCA revealed transcriptomic profiles are defined by region- and sex-specific variation (Supplemental Figure 4A). Thus, we completed downstream DEG comparisons in a region-and sex-specific manner.

### Cocaine-seeking ensembles in the NAc dynamically alter GPCR signaling and drd4 expression

Using neuronal deconvolution incorporating striatal single-cell RNAseq datasets as reference to compare with our bulk NAc RNA-seq data, we revealed all sample populations in the female NAc have the strongest proportion of D1 neurons while showing less proportion for D2 and interneuron (IN) neuron types (Fig. 2a,b). Further subcluster analysis revealed D1 neuronal subtypes defined by gene markers were similarly represented in each sample type. Specifically, within multiple D1 subclusters we saw differing neuronal subtypes featuring *Tac1* as a gene marker (Fig. 2c), a gene linked with substance P production and primarily involved with dopamine neurotransmission [17]. Within D2 subclusters (Fig. 2d) we saw differing neuronal subtypes featuring *Penk* as a gene marker, a gene linked with enkephalin production that is primarily used for endogenous opioid signaling and regulating cannabis exposure [18]. Supporting a previous study, it has been shown that cocaine exposure leads to an increase in the expression of both of these genes, *Penk* and *Tac1* [19]. Additionally, when looking into the enriched IN subclusters (Fig. 2e) we again saw subtypes defined by *Penk* similar to D2 while other subtypes were defined by *Syt1* expression, a key regulator of neurotransmitter release with evidence of downregulated expression leading to increased alcohol-associated behavior [20], and *Gad1* expression, the primary source of GABA production with previous neuropathogenic implications [21].

To provide comprehensive insight into how cocaine-seeking ensembles relate to sucrose-seeking ensemble, overlapped ensemble, and non-ensemble population within the female NAc, we used multiple DEG analysis to compare: 1) cocaine-seeking and the non-ensemble population 2) cocaine- and sucrose-seeking ensembles directly 3) cocaine-seeking and the overlapping ensemble. Several hundred genes were significantly (FDR < 0.01) up (log2FC > 0.75) or downregulated (log2FC < 0.75) for each DEG comparison with the cocaine-seeking ensemble (Fig. 2f). Combining the DEG from each of these comparisons with the cocaine-seeking ensemble, we found histone-coding genes [22] (upregulated: *H2al1e, H2ac24;* downregulated: *H2bc23*) that were similarly expressed when comparing cocaine-seeking to the non-ensemble or sucrose-related seeking ensembles. Histone-coding genes are directly associated with altering the epigenetic after chronic drug intake to facilitate long-term drug seeking [23]. Supporting the relevance to cocaine use disorder, *H2al1e, H2ac24* and *H2bc23* genes are all associated with the “Alcoholism” Kegg pathway (#ko05034) [24].

To further detect gene expression patterns related to the cocaine-seeking ensemble when compared to the sucrose-seeking ensemble or the non-ensemble, we used a Reactome gene ontology analysis to reveal enrichment of multiple GPCR-related pathways (Fig. 2g). Assessing the averaged log2FC of DEG from the cocaine-seeking ensemble when compared to each reference, we saw dynamic gene expression related to cocaine-seeking within the “GPCR ligand binding” gene interaction network (Fig. 2h). GPCR are canonically known to regulate addictive phenotypes for multiple drug classes including cocaine [25,26]. Specifically, *Gpbar1*, a gene shown to have a functional role in neuronal synapse degeneration through increasing opioid-binding cell adhesion molecule (*Opcml*) [27], was upregulated the most within this pathway for cocaine-seeking (Fig. 2i). *Drd4* from the dopamine receptor family was also upregulated (Fig. 2i), and has been annotated to facilitate a role within drug relapse [28–30]. Additionally, *Galr3*, a gene coding for a neuropeptide galanin receptor linked with drug addiction [31,32], was upregulated in the cocaine-seeking ensemble (Fig. 2i).

When looking into cell deconvolution within the male NAc, we saw, similarly to females, that the majority of the samples defined as D1 type MSNs with *Tac1* expression marking the enriched subclusters (Fig. 2j–l). D2 subcluster analysis highlighted enrichment of a *Penk* cell type (Fig. 2m) while interneuron subclusters were enriched the most for *Syt1/Gad1* cell type (Fig. 2n). Determining the DEGs of the cocaine-seeking ensemble by comparison with the sucrose-seeking ensemble, overlapping ensemble, or non-ensemble as the reference in the male NAc, we saw several hundred genes up or downregulated (Fig. 2o). Observing the extremities of DEG analysis between cocaine-seeking and multiple references revealed *Pde2a* expression was upregulated (Fig. 2p). To support the drug-linked relation of upregulated Pde2a within cocaine-seeking ensembles, previous work has shown inhibition of *Pde2a* decreases PTSD-induced alcohol consumption through the regulation of cAMP [33]. Additionally, *Pde2a* expression regulates exploratory behavior through NOS-mediated mechanisms [34] which separately have been linked to facilitating cocaine-induced behavior [35]. Assessing gene ontology pathways within the male NAc cocaine-seeking ensemble, we again, like in the female NAc, saw enrichment of the “GPCR ligand binding” pathway (Fig. 2q). Unlike the female NAc, the expression of cocaine-seeking ensembles for gene interactions within this pathway revealed *Drd4*, a previously highlighted drug-linked gene, was downregulated (Fig. 2r). In summary, the DEG results presented exhibit cocaine-seeking ensembles in the NAc have meaningful transcriptomic differences when compared to sucrose-seeking and the non-ensemble population.

### Cocaine-seeking ensembles in the mPFC have differential expression of histones and are linked with genes shown to decrease drug-linked behavior

**Fig. 3.**
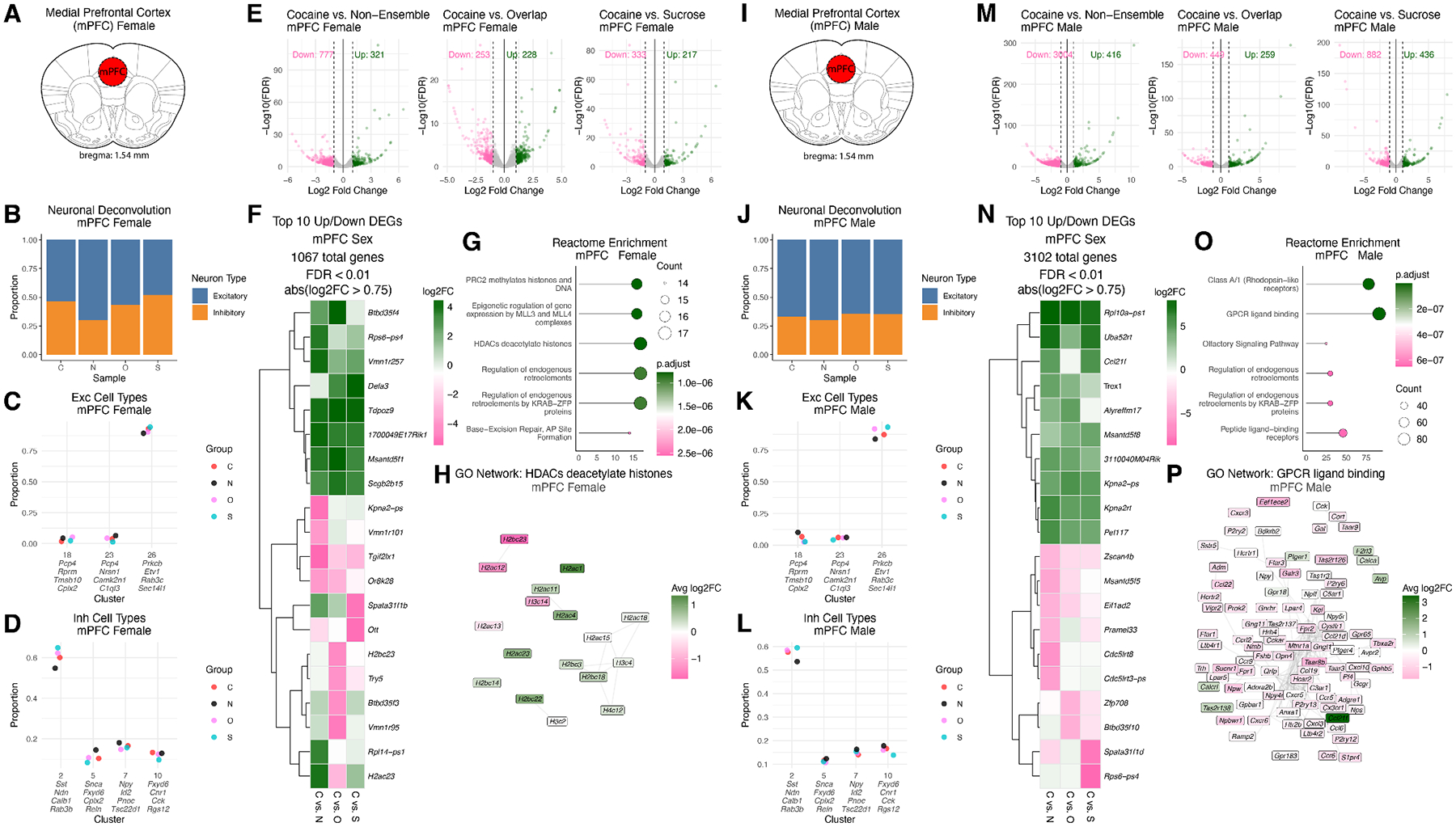
Molecular and cellular landscapes of cocaine-seeking ensembles in the medial prefrontal cortex (mPFC). **A,I** Atlas schematic showing the mPFC dissection site used for bulk RNA-seq in female (**A**) and male mice (**I**; mice per sex: *n* = 9 mice grouped into 3 replicates of 3-pooled mice; see Supplemental Figure 1 for full experimental workflow). **B,J** MuSiC-based deconvolution of bulk RNA-seq showing estimated proportions of excitatory and inhibitory neurons across ensemble-defined groups: cocaine-seeking ensemble (C), non-ensemble (N), overlapping ensemble (O), and sucrose-seeking ensemble (S) - in female (**B**) and male (**J**) mPFC. **C,D,K,L** Refined deconvolution results for excitatory (**C, K**) and inhibitory (**D, L**) neuronal subclusters in female (**C,D**) and male (**K,L**) mPFC. Each point reflects estimated proportions per cluster across groups; cluster labels include top four marker genes by log_2_ fold change. **E,M** Volcano plots showing differentially expressed genes (DEGs) after comparing the cocaine-seeking ensemble with the non-ensemble, sucrose-seeking, or overlapping ensemble as the reference in female (**E**) and male (**M**) mPFC. Genes meeting FDR < 0.01 and the absolute value (abs) of log_2_FC > 0.75 are colored (hot pink: downregulated, dark green: upregulated) while the genes that did not meet the criteria are grey. **F,N** Heatmaps showing the top 10 upregulated and downregulated DEGs per contrast in female (**F**) and male (**N**) mPFC, based on log_2_ fold change. **G,O** Dot plots showing Reactome pathway enrichment of DEGs combined from all of the criteria-met DEG from each comparison in female (**G**) and male (**O**) mPFC. Dot size reflects the number of DEGs per pathway; color indicates adjusted p-value. **H,P** GO network diagrams highlighting the top enriched Reactome pathway in female (**H**) and male (**P**) mPFC. Genes are colored by average log_2_ fold change (dark green = upregulated; hot pink = downregulated).

For neuronal deconvolution in the female mPFC, we incorporated cortical single-cell RNA-seq datasets as reference to compare with our bulk RNA-seq data. We revealed all sample populations in the mPFC (Fig. 3a) have the strongest proportion of excitatory neurons compared to inhibitory neurons (Fig. 3b). Further subcluster analysis revealed there are excitatory neuronal subtypes defined by gene markers similarly represented in each sample type (Fig. 3b). Specifically, the most enriched excitatory neuronal subcluster was defined by *Prkcb* expression (Fig. 3c) which has previously been classified within the genetic signature of heroin addiction [36] and other neuropathological disease-types [37]. Within inhibitory subclusters (Fig. 3d), the most enriched neuronal subtype was marked by *Sst* expression, a gene regulating somatostatin production that mediates neuromodulation after morphine or alcohol exposure [38–40].

Comparing the cocaine-seeking ensemble to the sucrose-seeking ensemble, overlapping ensemble, or the non-ensemble population as references resulted in several hundred significant DEGs in the female mPFC for each reference comparison (Fig. 3e). Extremities of the combined DEG from each reference comparison to the cocaine-seeking ensemble displayed robust expression of H2-type histone coding genes (*H2ac23* and *H2bc23*) associated with “Alcoholism” as highlighted in the female NAc (Fig. 3f). However, unlike the female NAc where *H2ac23* and *H2bc23* were expressed similarly between each cocaine-seeking and reference DEG comparison, the female mPFC had dynamic expression depending on the DEG reference. Specifically, *H2ac23* was upregulated when comparing the cocaine-seeking ensemble to the non-ensemble and the sucrose-seeking ensemble, but was downregulated when compared to overlapping ensemble. *H2bc23* was also only downregulated for the cocaine-seeking ensemble when compared to the overlapping ensemble, but featured minimal differential expression when compared to the non-ensemble population and the sucrose-seeking ensemble.

To further characterize cocaine-seeking DEGs in the female mPFC, we utilized the Reactome database to identify enriched transcriptomic networks (Fig. 3g). Considering the previously highlighted alterations in histone expression related to cocaine-seeking in the female NAc, we observed significant enrichment of the “HDACs deacetylase histones” network which have largely been associated as major contributors to facilitating drug addiction [23]. When looking into the gene interaction network for “HDACs deacetylase histones”, we can again appreciate the dynamic expression of histones within the female mPFC, e.g., *H2ac1, H2ac4, H2ac22,* and *H2ac23* were upregulated robustly while *H2bc23, H2ac12*, and *H3c14* had were downregulated (Fig. 3h).

Within the male mPFC (Fig. 3i), all samples were defined by enrichment of excitatory cell types while again (Fig. 3j), deeper subcluster analysis revealed enrichment of *Prkcb* (Fig. 3k), a gene previously highlighted in the female mPFC to be associated with heroin addiction [36], excitatory cell type. We again saw enrichment of *Sst* inhibitory cell types as seen with the female mPFC (Fig. 3l). Utilizing the same approach as our other DEG analyses, we compared the cocaine-seeking ensemble with the non-ensemble, sucrose-seeking ensemble, or overlapping ensemble as the reference to reveal several hundred significant DEGs (Fig. 3m). Highlighting the extremities of DEGs related to cocaine-seeking in the male mPFC (Fig. 3n), we observed genes sharing direction of expression, such as *Pet117*. Specifically, *Pet117* is annotated with regulating mitochondrial function linked with neurological regression of motor skill development and as an indicator for pyramidal signs, i.e., cortical dysfunction [41].

Assessing enrichment of gene ontology within the male mPFC, we saw again, like the female and male NAc, enrichment of the “GPCR ligand binding” pathway (Fig. 3o). Observing the expression of combined cocaine-seeking DEGs in this pathway (Fig. 3p), we saw an overexpression of *Avp*, the gene encoding for the dopamine-mediating vasopressin precursor [42]. Antagonism of *Avp* receptors has been linked with decreasing morphine conditioned place preference [43]. Additionally, *Taar8b* from the trace-amine associated receptor (TAAR) family, was downregulated in the cocaine-seeking ensemble. TAAR family genes have been shown to reduce cocaine seeking without affecting sucrose intake after receptor agonism [44]. Together, these multifaceted DEG comparisons establish that cocaine-seeking ensembles in the mPFC have unique gene expression that is separate from sucrose-seeking and the non-ensemble population.

### Ensemble-specific weighted gene co-expression network analysis

**Fig. 4.**
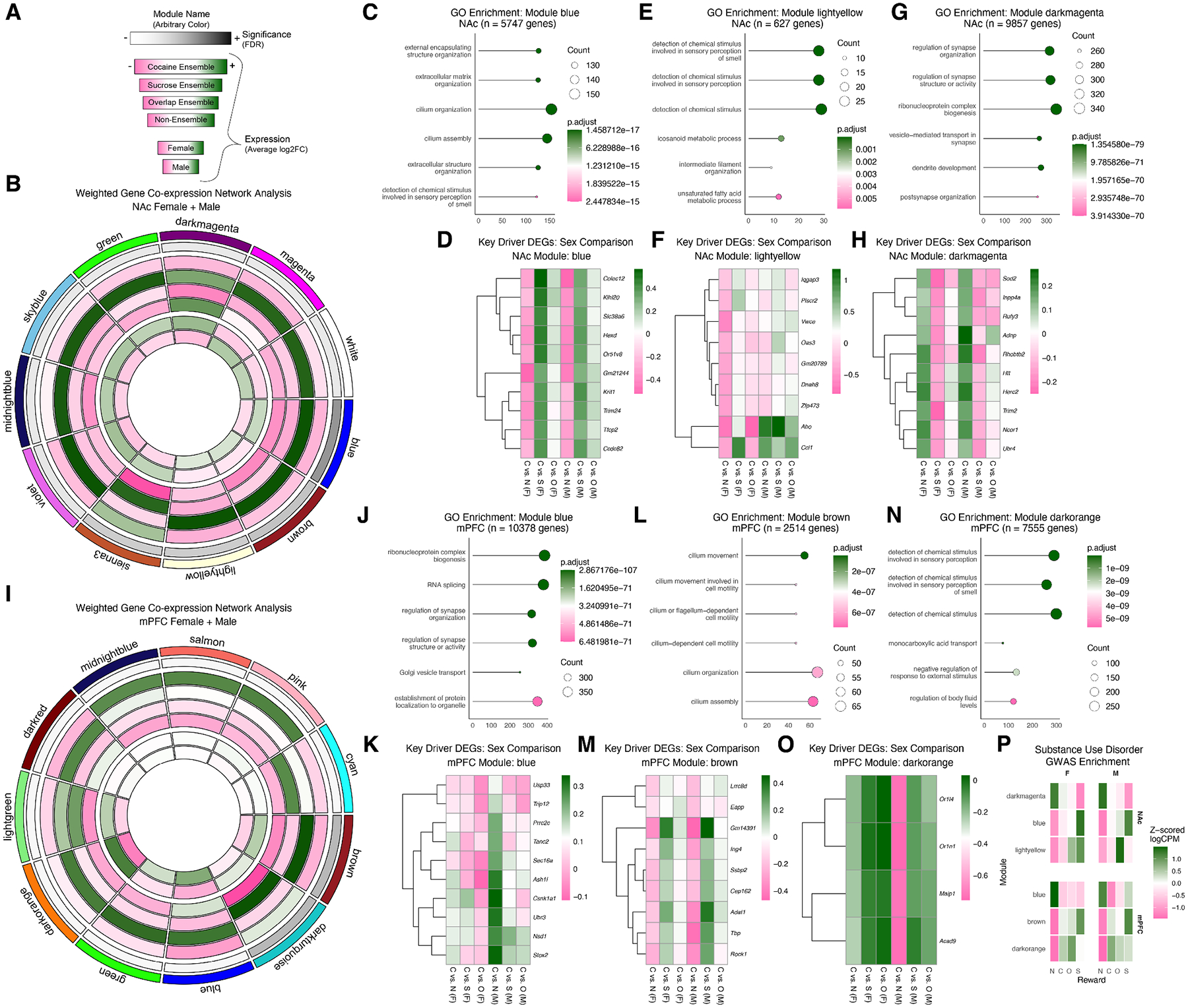
Identifying transcriptional programs within cocaine-seeking ensembles. **A** Key for weighted gene co-expression network analysis (WGCNA) showing the relationship between module eigengenes and experimental conditions (ensemble, region, and sex) in NAc (**B**) and mPFC (**I**). **B,I** Circular barplots summarizing all modules identified by WGCNA across female and male samples in NAc (**B**) and mPFC (**I**). Ring order follows the key in **A. C,D,G** Reactome gene ontology (GO) enrichment analysis for selected modules in NAc: blue (**C**), lightyellow (**E**), and darkmagenta (**G**). Dot size reflects the number of genes per pathway; color indicates adjusted p-value. **D,F,H.** Heatmaps of key driver genes from NAc modules blue (**D**), lightyellow (**F**), and darkmagenta (**H**), comparing average expression (log_2_FC) across reward groups and sex. **J,L,N.** GO enrichment for selected mPFC modules: blue (**J**), brown (**L**), and darkorange (**N**), shown with dot size (gene count) and color (adjusted p-value). **K,M,O** Heatmaps of key driver genes from mPFC modules blue (**K**), brown (**M**), and darkorange (**O**), visualizing average expression (log_2_FC) across reward groups and sexes. **P** Enrichment of human substance use disorder GWAS-mapped genes within key WGCNA modules, stratified by ensemble (N, C, O, S) and brain region (NAc, mPFC). Each tile represents the relative expression (z-score) of substance use disorder GWAS genes in a given module and condition. Green color indicates higher enrichment while pink color indicates lower enrichment relative to the mean of that module across groups.

For detecting transcriptomic patterns using an unsupervised and unbiased approach, we utilized gene co-expression network analysis (WGCNA) [45] to compare cocaine-seeking, sucrose-seeking, and overlapping ensembles in addition to the non-ensemble population while considering sex as a variable in the NAc or mPFC. The modular analysis from the WGCNA analysis in the NAc resulted in modules with differential expression patterns between each ensemble type and the non-ensemble (Fig. 4a,b). Observing the gene ontologies enriched within the “blue” module in the NAc resulted in numerous pathways associated with cilium or the extracellular matrix (Fig. 4c). Recent work has implicated cilia-mediated mechanisms related to reducing cocaine induced locomotion and conditioned place preference while also aiding in tolerance to morphine [46,47]. To determine genes within this “blue” NAc module that direct a large number of downstream gene expression patterns, we performed key driver analysis. We then associated these key drivers with sex-specific gene expression profiles from previous DEG comparisons of the cocaine-seeking ensemble with multiple reference combinations (Fig. 4d). This analysis revealed the expression of key drivers for the cocaine-seeking ensembles when compared to the sucrose-seeking ensembles were upregulated in contrast to downregulation when the cocaine-seeking ensemble is compared to the non-ensemble. *Colec12* has been implicated in a GWAS associating single nucleotide polymorphisms within genetic addiction data from humans [48]. Further, *Slc38a6*, a glutamine transporter, expression is shown to be altered after prolonged methamphetamine exposure [49].

Looking into the GO enrichment of the “lightyellow” module in the NAc resulted in enrichment of the “detection of chemical stimulus” and “intermediate filament organization” pathways which support the idea that stimuli-based memories [50] are dictated by rearrangement of cytoskeletal anatomy within neurons to form established ensembles dictating addictive phenotypes (Fig. 4e) [51]. Assessing the key drivers for the lightyellow module resulted in mostly genes that were enriched in the female cocaine-seeking ensemble when compared to the non-ensemble reference, such as downregulation of *Oas3*, a gene implicated in an alcohol consumption GWAS (Fig. 4f) [52]. Further, we saw downregulation of *Zfp473* which has been associated with genetic studies for cocaine IVSA [53].

Assessing the enrichment of the “darkmagenta” module resulted in enriched pathways associated with synaptic function including “regulation of synapse organization” and “regulation of synapse structure or activity” (Fig. 4g), which are canonical to providing the signaling effort required to elicit drug craving [54]. Looking into key drivers regulating the “darkmagenta” module (Fig. 4h), we saw mostly upregulation in the cocaine-seeking ensemble when compared to the non-ensemble in contrast to downregulation of genes when comparing cocaine-seeking and sucrose-seeking directly. Specifically, *Trim2*, a gene implicated in a GWAS for Parkinson’s disease [55] followed this pattern of gene expression dependent on the reference population. Parkison’s disease is more likely to develop for those who use chronic cocaine with predisposing genetic susceptibility for addiction [56]. Further, *Sod2* also followed this pattern of gene expression within this module and has previously been linked with regulating the decreasing presence of gray matter after chronic alcohol exposure [57].

Similar to the NAc, the top 10 modules from the WGCNA analysis in the mPFC (Fig. 4i) resulted in modules with dynamic expression patterns related to cocaine-seeking, sucrose-seeking, and overlapping ensembles, and the non-ensemble population. The “blue” module in the mPFC showed enriched gene ontology pathways associated with cellular morphology linked with synaptic organization, structure, and function for vesicular trafficking, previously highlighted in the NAc (Fig. 4J). Assessing the genes determined as key drivers for the “blue” mPFC module, we can appreciate dynamic expression with a definitive pattern of upregulation of genes when comparing the cocaine-seeking ensemble with the non-ensemble population for males (Fig. 4k). Specifically, *Stox2* was upregulated in this manner for male cocaine-seeking compared to the non-ensemble. *Stox2* has been strongly associated in a GWAS of impulsive personality traits [58], a hallmark trait for those with CUD [59]. *Prrc2c*, which was only upregulated in the male mPFC when comparing cocaine-seeking to the non-ensemble population, has been shown to have altered expression after chronic cocaine self-administration [60], correlating highly with our study.

Similar to enrichment of cilium related cellular mechanisms, the “brown” module in the mPFC was also enriched for pathways annotated for cilium assembly, organization, and motility (Fig. 4l). Within the key driver analysis for the “brown” module, we found the gene *Cep162* was upregulated for the female and male cocaine-seeking ensemble when compared to the sucrose-seeking ensemble in contrast to when compared to the non-ensemble population where *Cep162* was downregulated (Fig. 4m). *Cep162* is involved in ciliary function and has been previously shown to regulate cellular function during alcohol and morphine withdrawal [61,62]. Additionally, *Adat1* also followed this gene expression pattern as *Cep162* and has been shown to be downregulated after treatment to reduce methamphetamine intake [63].

When looking into the “darkorange” module, we found enrichment of pathways related to “detection of chemical stimulus” and the “negative regulation of response to external stimulus” (Fig. 4n). Observing the genes within the “darkorange” pathway revealed only downregulation in the males when comparing the cocaine-seeking to the non-ensemble population (Fig. 4o). Specifically, *Maip1* and *Acad9* have been linked to neurological pathologies in previous studies [64,65].

### Cocaine-seeking features gene expression modules regulating stimuli detection that correlate with human GWAS

To evaluate whether transcriptional modules identified in the WGCNA analysis were enriched for genetic risk factors of substance use disorders (SUD) in humans, we utilized a publicly available SUD GWAS gene set where 245 SUD-related GWAS were combined to compare with our mouse bulk RNAseq WGCNA module gene set for each region- and sex-specific ensemble or non-ensemble population (Fig. 4p). Within the NAc, the enrichment analysis characterized the “darkmagenta” module to be most enriched with the non-ensemble population for each sex. The NAc “blue” module correlated the most with the sucrose-seeking ensemble for both sexes. The “lightyellow” module was highly enriched with the overlapping ensemble in the males and while we saw enrichment for both the sucrose-seeking and overlapping ensemble in females. Assessing GWAS gene set enrichment analysis within the mPFC, we saw the “blue” module enriched in the non-ensemble population for both sexes. The “brown” module was mostly enriched for the sucrose-seeking ensemble while the “darkorange” module was highly enriched in the overlapping ensemble for females and strongly enriched in the cocaine-seeking ensemble within males.

## Discussion

We used within-subject cocaine and sucrose dual SA conditioning followed by extinction training and cued reinstatement to induce cocaine and sucrose seeking in cFos-TRAP2 mice (Fig. 1) following procedures in our previous study [7]. Through FACS, we isolated neurons from reward-specific seeking ensembles and the non-ensemble population for downstream transcriptomic profiling within two critical reward-signaling regions, the NAc and mPFC. We then used an unbiased approach to identify gene expression signatures related to the region- and sex-specific cocaine-seeking ensembles when compared to the sucrose-seeking ensembles, overlapping ensembles, or the non-ensemble population as references.

### Sex differences in cocaineand sucrose-seeking behavior and underlying transcriptome

Although identifying minimal sex differences in behavioral training, it should be considered that we did not record the estrous cycle in this study which is known to influence cocaine-seeking [66] and food rewards [67] in female rats. Further, the principal component analysis revealed an effect of sex on gene expression which supports previous studies showing cocaine exposure leads to sex-specific transcriptomic alterations [68]. Thus, we completed downstream DEG comparisons in a sex-specific manner.

### Within-subject cocaineand sucrose-ensemble identification, isolation, and characterization

In this study, we combined TRAP2 technology [15] during cocaine-seeking behavior with immunolabeling during sucrose-seeking behavior to identify reward-seeking ensembles tagged at different time points. While both approaches use c-fos expression as a proxy for neuronal activation [4], we acknowledge that not counterbalancing the tagging method, i.e., tagging the sucrose-seeking ensemble with TRAP and the cocaine-seeking ensemble with c-Fos immunolabeling, limits the interpretation of the results. However, we previously showed that cocaine-seeking ensembles tagged with these two techniques during cue-induced reinstatement of cocaine overlap by 50 % [7], which is a similar reactivation level to what has been reported in the literature [15, 69–71]. Moreover, when counterbalancing TRAP and immunolabeling approaches to tag cocaine- or sucrose-seeking ensembles, the percentage of overlap between ensembles was up to 30 % independent of the tagging method used.

Furthermore, from our flow cytometric results, we determined the size of female and male cocaine-seeking, sucrose-seeking, and the overlapping ensembles had a measurable difference for tagged ensemble size that was dependent on ensemble type and region where the sucrose-seeking ensemble was larger (Supplemental Figure 3L). We suggest the difference in ensemble size could be due to variability in the tagging process where the sucrose population will always have more active neurons due to sorting based on endogenous Fos expression compared to TRAP2 labeling of the cocaine-ensemble which is not necessarily active during the sorting process. This does not discount the bioinformatic analysis using the presented order of seeking events despite a lack of counter tagging control that we acknowledge is of great interest. Rather, interpretation of results should be in the framework of comparing the persistent effects of neurons previously activated during cocaine-seeking in comparison to the real-time processing for natural (sucrose) reward seeking. Indeed, considering the size differences in ensembles, it is important to note that we normalized ensemble sizes by sorting an equal number of events for each reward-seeking ensemble and used downstream bioinformatic approaches to normalize for the effect of fragment read counts that could be influenced by ensemble size.

Additionally, it should be acknowledged that the tagging framework for cocaine- and sucrose-seeking ensembles captures gene expression spanning the entirety of the reinstatement session rather than having resolution to specific behavior elicited during the seeking session. Further, it is known that both cocaine and sucrose act as exogenous reinforcers and induce distinct physiological states [72,73]. Cocaine primarily amplifies motivation and drive to seek cocaine, whereas sucrose more directly satisfies homeostatic needs because of the caloric value. In this context, our FACS-based ensemble analysis was designed not to isolate pharmacological effects per se, but to capture the neuronal populations recruited during the entire reward-seeking session. This approach allows us to identify where cocaine-driven motivational gene expression programs converge with, or diverge from, homeostatic reward mechanisms. Furthermore, the presence of an overlapping population of neurons identified between cocaine- and sucrose-associated ensembles in a previous study [7] using immunohistochemical techniques, in addition to this study using bioinformatic techniques, suggests that cocaine seeking co-opts neural substrates normally engaged in homeostatic-driven reward behaviors. We hypothesize that with chronic cocaine exposure, the brain begins to treat cocaine-associated cues as indicators of a physiological need even though these cues do not necessarily contribute to restoring homeostatic balance.

To further support our ensemble tagging approach, we quantified the enrichment of IEGs in comparison to the non-ensemble to determine levels of activity-dependent mRNA (Supplemental Figure 5). In the NAc and mPFC samples for both sexes, IEG expression is mostly exclusive to the sucrose-seeking ensemble population. It should be noted that tagging sucrose-seeking neurons relies on cFos protein expression rather than mRNA, and that IEGs have varying times of peak mRNA expression and decay rates with some genes (*Fos*) having shorter (~30 min) window of expression [74] than others (*Arc, Npas4*) that reach peak expression after longer periods (~60 min) [75,76]. Further assessing sample variability (Supplemental Figure 4), we acknowledge variation between replicates but highlight the limitation of pooling samples from mice with varying levels of self-administration, extinction, or seeking behavior, which was required to have an adequate amount of RNA for downstream bulk RNA sequencing We did not have the statistical power to determine behavioral stratification of cocaine- and sucrose-seeking to correlate with transcriptomic profiles. This approach introduces higher variation from pooled replicate to replicate (Supplemental Figure 4B) compared to using transcriptome samples from individual mice that can be correlated with behavior, which is more commonly seen within non-FACS bulk RNAseq or single-nuclei RNAseq approaches, where tissue input parameters differ. However, statistical assessment to determine differences between pooled-sample replicates based on their seeking behavior proved to be non-significant (Supplemental Table 1g), thus justifying combining pooled samples as replicates for downstream bioinformatic processing. Altogether, we propose that combining TRAP and immunolabeling approaches for tagging and extracting ensembles within the same subject is of value and that the genes identified to be differentially expressed in the cocaine-seeking ensemble are relevant to finding targets that are likely specific to cocaine seeking behavior without altering that of sucrose seeking.

### Cell deconvolution within Cocaineand sucrose-seeking ensembles

Since our earlier study using the same dual cocaine and sucrose paradigm showed that cocaine-seeking ensembles recruit a majority of D1-MSNs within the NAc [7], similar to what was previously reported in the literature [77–79], it is rational to hypothesize that most of the differential gene expression reported in the cocaine-seeking ensemble might arise from changes in D1-MSNs. Using neuronal deconvolution revealed the majority of neuronal enrichment presented as D1-MSN cell types in all the reward-seeking ensembles and non-ensemble population within the NAc (Fig. 2). Further supporting the literature [80], most cell types in the mPFC from the cell deconvolution analysis or our samples were deemed excitatory cell types (Fig. 3). These analyses provide evidence that the cell type composition within our bulk RNAseq samples are relevant to established literature.

### Cocaine-seeking ensembles feature dynamic gene expression of previously studied SUD-related gene candidates

Compared to the non-ensemble population of neurons not involved in cocaine or sucrose seeking, we identified significant (FDR < 0.01) ensemble-specific transcriptomic alterations (log2FC > 0.75; log2FC < −0.75) in cocaine-seeking when compared to the sucrose-seeking ensemble, overlapping ensemble, or the non-ensemble population as reference. Observing the extremities of reward-specific gene expression in male and female NAc and mPFC revealed patterns of gene expression likely related to cocaine-seeking when compared to the multiple references (Fig. 2,3). These results suggest that there are robust transcriptomic changes driving cocaine-seeking that are different from the non-ensemble population and sucrose-seeking ensemble.

Specifically, we found distinct alterations in gene expression of histone coding genes in the female NAc and mPFC. Histone-mediated epigenetic modifications are believed to underlie the persisting effects of addictive behavior by leaving long-lasting influence onto the genomic landscape [23]. This supports the idea that drug relapse is driven by the persisting molecular effects created during intake which outlast abstinence to facilitate seeking behavior [81]. Moreso, the specific histones enriched in our data set coincide with those annotated previously in the “alcoholism” gene ontology pathway, suggesting a common pathway of epigenetic modification within multiple addictive drug classes. Within the male NAc, we also saw overexpression of *Pde2a* which has been functionally tested to reduce stressed induced alcohol consumption [33]. This is a good indicator that inhibition of *Pde2a* could possibly reduce cocaine-related behavior. For both the female and male NAc, we saw upregulation of *Galr3* expression, a gene that has been established to use galanin-mediated pathways for regulating dopamine neurotransmission [31,32]. We saw differing directions of *Drd4* expression between females and males in the NAc, with upregulation in the females and downregulation in the males. Strikingly, *Drd4* expression has been linked to have sex-dependent outcomes for seeking behavior [82] in addition to being considered a factor for regulating increased motivation both for sexual interactions [83] and risky financial behavior [84]. This supports the idea that cocaine-seeking ensembles utilize motivational reward-attaining strategies to elicit strong cocaine-seeking behavior. We also saw “GPCR ligand binding” as one of the top enriched pathways in all of our DEG comparisons except the female mPFC. GPCRs are canonically linked to signaling mechanisms between cells, especially for neurotransmission, which is a hallmark for carrying out cocaine-seeking behavior [25,53]. Overall, we identified numerous gene candidates throughout the DEG analysis that have been previously shown to have a role in altering drug-linked behavior or have been a product of drug exposure itself (*H2al1e, H2ac24, H2bc23, Gpbar1, Drd4, Galr3, Pred2a, Avp,* and *Taar8b*). It is likely that targeting these candidates could lead to exclusive decrease in cocaine-seeking behavior without disturbing sucrose-seeking behavior.

### Cocaine-seeking ensembles are defined by dynamic expression within synaptic, stimuli-detecting, and ciliary networks in corticostriatal regions

Although we found exciting candidates in our DEG approach, we felt it was necessary to use an unbiased approach to examine transcriptomic signatures in each region while considering sex by letting the expression from each sample type dictate the enriched genes rather than explicitly stating which DEG comparisons to make. After reviewing several enriched modules, we identified enrichment of cilia related pathways within both regions. Considering previous studies on ciliary mechanisms in regulating drug-induced behavior [61,62], this finding suggests a role for cilia in aiding the cross-talking network of neurons engaged to elicit cue-induced cocaine seeking. Further, we found enrichment of stimuli-detecting pathways within both brain regions underlining our cue-induced seeking paradigm and previous studies demonstrating that drug seeking is reinforced by once salient environmental cues paired initially during drug intake [85]. We also found enrichment of pathways related to synaptic regulation in both regions which is canonically known to facilitate drug-seeking behavior [86]. Overall, we found many key drivers (*Colec12, Slc38a6, Oas3, Zfp473, Sod2, Prrc2c,* and *Cep162*) within these pathways with robust expression for cocaine-seeking that have been previously associated with drug-linked behavior, thus suggesting the importance of studying these genes in future investigations.

Preclinical research for substance use disorder using models that induce drug intake and seeking behavior are only useful in the scope for how they translate into genotypes underlying human SUD. To address this, we used enrichment with genome wide association studies (GWAS) in humans to assess the enrichment of human SUD-related genes in relation to the WGCNA modules enriched in the cocaine-seeking ensemble, sucrose-seeking ensemble, the overlapping ensemble, and the non-ensemble population in a mouse model for drug and non-drug seeking. We found enrichment of human SUD genes for the cocaine-seeking and overlapping ensemble in a module that was defined by gene ontology related to detection of external stimuli which further highlights the importance of addressing neurobiological mechanisms related to cue-reinforced drug taking behaviors to prevent further drug relapse. Beyond this enrichment, we also found pathogenic genes in our DEG analysis that were found in human GWAS related to SUD (*Colec12* and *Oas3*), impulsive personality (*Stox2*), and Parkinson’s disease (*Trim2*). Together these results highlight that gene signatures linked with cocaine-seeking apart from sucrose-seeking that were identified by the preclinical model in this study do correlate with genetics underlying human traits of SUD. However, this also highlights the complexity of gene expression when trying to detangle reward-seeking ensembles for drug and non-drug rewards.

## Conclusion

Taken together, this investigation has identified, isolated, and characterized gene expression patterns related to cocaine-seeking ensembles that are likely separate from those genes involved in the sucrose-seeking ensemble or the non-ensemble population. This repository of reward-specific transcriptomic information is a valuable resource for further functional validation of target genes to reduce cocaine-seeking without altering other types of reward seeking behavior.

## Materials and methods

### Ai14:cFos-TRAP2 mice

Female and male Ai14:cFos-TRAP2 transgenic mice were obtained by crossing male tamoxifen-inducible cFos- Cre-recombinase knock-in mice (Fostm2.1(icre/ERT2)Luo/J, Strain# 030,323; The Jackson Laboratory) with female Ai14 loxP-flank regulated Cre-reporter knock-in mice (B6;Cg-Gt(ROSA)26Sortm14(CAG-tdTomato)Hze/J; Strain# 007,914; The Jackson Laboratory). Mice were individually housed on a 12:12 reverse light schedule during dual-reward self-administration conditioning, as described previously [7] All procedures were conducted in accordance with the IACUC and NIH animal handling procedures.

### Dual reward self-administration, extinction, and reinstatement tests

Female and male 8–16-week-old mice (20–30 g) underwent catheter implantation according to [7]. After recovery, mice underwent dual-reward self-administration of intravenous cocaine infusions (0.5 mg/kg/inf; NIDA) and oral intake of sucrose pellets (15 mg; Bio-Serv) during 2-hour daily sessions (10 days of cocaine reward and ten days of sucrose reward: 20 alternating days in total) in standard mouse modular test chambers (Imetronic, France). Each chamber included two nose-poke (NP) holes associated with cocaine infusions or delivery of sucrose pellets. Each NP was paired with an availability light cue (always on until mice associate with NP; off for ten seconds after NP activation) and an internal NP light cue (on for 3 s after NP activation). The reward-associated NP not used during a reward-specific session served as an inactive NP (i.e., cocaine-associated NP during a sucrose session acted as an inactive NP with no cues or reward administration and vice versa). During self-administration acquisition, all mice were on a food-restricted diet (80 % of average food intake = 3.2 g) to increase reward-specific operant conditioning responses [87,88]. Following reward-specific cue conditioning, mice were extinguished of rewards and cues until reaching a 70 % reduction of NP association compared to the averaged last three days of reward self-administration. Following the extinction phase, 30-minute reinstatement sessions for each reward were carried out with the reward-associated light cue but without administering the reward. Following intraperitoneal injection of 4-hydroxy-tamoxifen (4-OHT, 50 mg/kg, Sigma) directly after the cocaine reinstatement session, the Cre-regulated TRAP2 mechanism tagged active cocaine-seeking cells with endogenous tdTomato fluorescence. After 4-OHT administration, mice were placed back in their home cage and were left undisturbed until the next extinction session the following day. Following sucrose reinstatement sessions, mice were euthanized 60 min post-session to capture peak cFos expression reflective of active cells during sucrose-seeking behavior.

### Extraction of reward-specific ensemble RNA

Whole brains were extracted from euthanized mice (5 % isoflurane exposure followed by cervical dislocation) and incubated for 5 min on ice in cellular stabilizing solution: 45 % HEBG medium (2 % 50X B27 (Thermo Fisher; 17,504,044) and 0.25 % 100X Glutamax (Thermo Fisher; 35,050,061) per 1 mL of Hibernate E-Minus Phenol Red (Thermo Fisher; NC1506837)), 45 % artificial cerebral spinal fluid (aCSF; 7.13 g/L NaCl (Sigma-Aldrich; S9888-500 G), 0.23 g/L KCl (Sigma-Aldrich; P3911–25 G), 0.14 g/L MgSO4 (Sigma-Aldrich; 746,452–500 G), 0.19 g/L CaCl2 (Sigma-Aldrich; C33067–100 G), 0.048 g/L NaH2PO4 (Millipore-Sigma; 10,049–21–5), 2.1 g/L NaHCO3 (Millipore-Sigma; 144–55–8), 1 L diH2O adjusted for dry reagent volumes), 9.97 % RNAlater (Thermo Fisher; AM7020), and 0.03 % RNaseOUT (Thermo Fisher; 10,777,019). Brain sections (1.94–0.64 bregma; 1.5 mm thickness) containing the nucleus accumbens and medial prefrontal cortex were cut using a brain matrix (0.5 mm spaced slits) kept cold at −20 °C with ice-cold razors. Punches of brain tissue were made using a 1 mm biopsy punch for each nucleus accumbens (primarily lateral accumbens shell and lateral accumbens core) bilateral region, and a 1.5 mm-sized punch for the medial prefrontal cortex (primarily prelimbic and cingulate areas 1 and 2). Brain regions from three mice per sex were pooled together for adequate RNA collection [89]. Brain punches were immediately placed in enzymatic dissociation solution (2mg/mL papain (Worthington; LS003120) diluted in 1 mL HEBG with 0.125 % 100x Glutamax – heated in 30C water bath for 10 min before immediately placing on ice for brain punches) on ice for 20 min. Mechanical trituration while incubating in papain solution was carried out using 10x syringe fluctuations with a 23 G needle followed by a 27 G needle. Caution is taken to not introduce air bubbles during syringe fluctuations that otherwise rupture cell membranes. Tissue was centrifuged for 3 min at 1000 rcf at 4 °C, and the supernatant was carefully discarded via pipette, leaving ~20 uL medium to prevent significant cell loss. Cells were slowly resuspended via pipette with 500 uL of 4 °C HEBG medium and then filtered through a 40 um cell strainer. Semi-permeabilization was carried out by adding 500 uL of −20 °C ethyl acetate (95 % ethanol; 5 % acetate) to the 500 uL of HEBG medium with cells for a final 50 % ethyl acetate concentration. Tubes were slowly inverted by hand for 1 min briefly before centrifuging for 5 min at 1000 rcf in 4 °C. The supernatant was discarded carefully via pipette leaving ~20 uL to prevent cellular loss and cells are resuspended gently via pipette in 500 uL of 4 °C HEBG medium with neuronal (NeuN-Alexafluor-405; conc. 5:500; Novus Biologicals; NBP1–92693AF405) and cFos-labeling conjugated antibodies (cFos-Alexafluor-488; conc. 5:500; Novus Biologicals; NBP2–50037AF488). Cells were incubated at 4 °C while rotating in the dark for 45 min. Cells were centrifuged for 5 min at 1000 rcf at 4 °C, and the supernatant was carefully discarded via pipette, leaving ~20 uL medium to prevent significant cell loss. Cells were resuspended in 1 mL of 4 °C HEBG medium and filtered with a 40 um cell strainer into a sterile 5 mL FACS tube and kept at 4 °C. Cells were then sorted for neuronal and reward-specific tags (neuron – immunolabeled NeuN; cocaine – endogenous tdTomato; sucrose – immunolabeled cFos; overlap – tdTomato/cFos) using FACS (FACSMelody; FACSChorus Software 100-um nozzle; BD Biosciences). Initial gating for NeuN-405+ cells was determined in comparison to a negative control using lysate from Ai14::TRAP2 mice lacking NeuN-405 staining. Using only NeuN-405+ population, subsequent quadrant gating for cFos-488+ (bottom right quadrant), tdTomato+ (top left quadrant), cFos-488+/tdTomato+ (top right quadrant), and cFos-488-/tdTomato-(bottom left quadrant), was determined using lysate from Ai14:: TRAP2 mice lacking 4-OHT and cFos-488 staining. Cocaine-seeking ensembles were determined with events featuring tdTomato+, sucrose-seeking ensembles were determined with events featuring cFos-488+, overlapping ensemble was determined with events featuring cFos-488+/tdTomato+, and non-ensemble population was determined with cells lacking cFos-488 or tdTomato signal. Only the first 1 million events were recorded to create the flow cytometric quadrant plots. 100k events (including cells and possible debris) per reward-specific population were sorted directly into the RLT buffer (RNeasy Mini Kit; Qiagen; 74,104) for downstream RNA extraction using RNeasy Mini Kit. RNA samples were kept at −80 °C until downstream RNA sequencing.

### RNA sequencing of reward-specific neurons

RNA samples underwent library preparation and next-generation RNA sequencing at the University of Colorado Anschutz Medical Campus Genomics and Microarray Core. Low-input ribosome depleted (SMART-seq; Takara Bio; library size: 200–500 bp) library prep was used for downstream paired-end 2 × 150 bp RNA sequencing at a 50 million read depth using NovaSEQ 6000 (Illumina). Raw fastq output files were used for downstream bioinformatic analyses. Raw fastq and transcript count files are available on gene expression omnibus (GEO) under accession number GSE247029.

### Bioinformatic analysis of reward-specific transcriptomes

Raw RNA-seq reads were adapter-trimmed using the Linux-based program CutAdapt [90] and quantified against the GRCm39 mouse transcriptome using Salmon [91]. Transcript-level estimates were summarized to gene-level counts with the “tximport” R package [92]. Gene-level counts were filtered for low expression, normalized, and fit to linear models using the “edgeR” (Robinson, McCarthy, and Smyth 2010) and “limma” [93] R packages. Reactome and Gene Ontology (GO) enrichment analyses were performed using the “clusterProfiler” [94] and “ReactomePA” [95] R packages. Cell-type deconvolution of bulk RNA-seq data was conducted using “MuSiC” [96], using an average of replicates per each sample type to compare with neuronal types and subtypes from striatal [97] or cortical [80] single-cell RNA-seq data sets. Differential expression of key neuronal subclusters was further assessed using “scran” [98]. Co-expression modules were identified using Weighted Gene Co-expression Network Analysis (WGCNA) [45]. Module-trait correlations were visualized and ranked using eigengene-trait association testing in “limma”, and GO enrichment for modules was visualized “ggplot” dotplots. Network plots were generated using “igraph” [99], “ggraph” [100], and “circlize” [101]. Expression of human substance use disorder GWAS (EBI MONDO_0002494) genes were matched to mouse gene homologs and were assessed across WGCNA modules by computing z-scored logCPM values within region, sex, and reward group.

### Statistical analysis

Behavioral data was analyzed using generalized linear mixed models (GLMMs) with a negative binomial distribution due to count overdispersion and lower AIC measure than other linear models. Models included fixed effects of reward, sex, session, and nose poke identity, with subject-level random effects to account for repeated measures. Separate models were fit for the self-administration, extinction, and reinstatement phases with post hoc estimated marginal mean pairwise comparisons with a false discovery rate (FDR) correction. For cell sorting data, the percentage of events within each FACS quadrant was analyzed using a linear model with fixed effects of condition, brain region, and sex, including all interactions. Significant main effects and interactions were followed by post hoc estimated marginal mean pairwise comparisons with FDR correction. Full behavior analysis is located in Supplemental Table 1. Differential gene expression analysis was performed using edgeR with TMM normalization and generalized linear modeling. Principal component analysis (PCA) was performed on normalized counts, and group differences were assessed using PERMANOVA and are reported in Supplemental Figure 4A. False discovery rate (FDR) correction was applied using the Benjamini–Hochberg method, and genes with FDR < 0.01 were considered significant. Full DEG analysis is located in Supplemental Table 2.

## Supplementary Material

1

2

3

Supplementary material associated with this article can be found, in the online version, at doi:10.1016/j.addicn.2025.100242.

## Data Availability

Data will be made available on request.
