# Supplementary material for: Corticostriatal cocaine-seeking ensembles are defined by differing gene expression from sucrose-seeking ensembles using a within-subject dual self-administration and seeking mouse model
Source: Addict Neurosci. Author manuscript; Available in PMC 2026 Jun 26. (PMC13298087; doi:10.1016/j.addicn.2025.100242)
Supplement: 2 [file NIHMS2186364-supplement-2.docx]

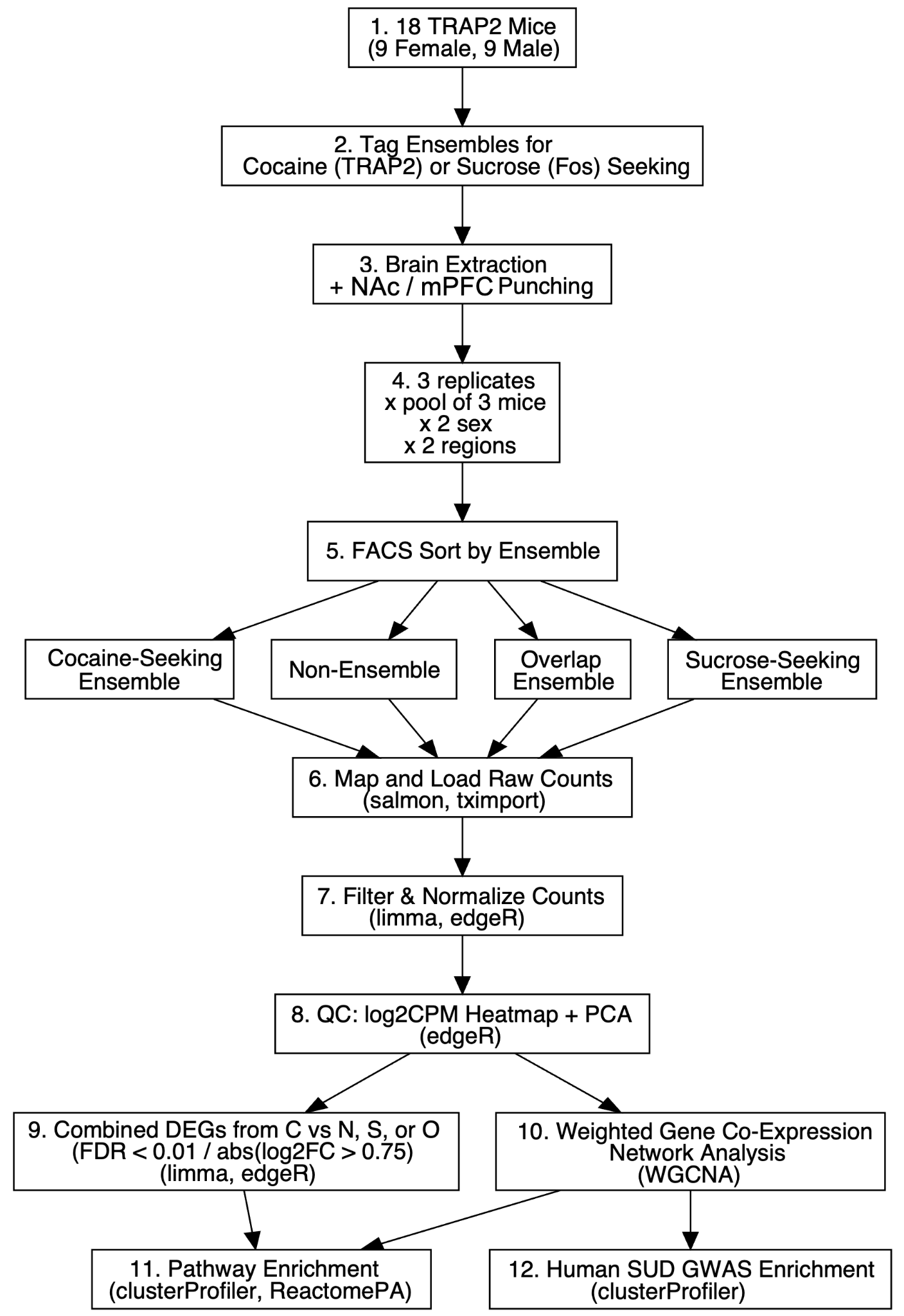


**Supplemental Figure 1. Experimental Approach.** **A** Schematic of experimental approach to capture cocaine and sucrose seeking ensembles and characterize the underlying transcriptomes.


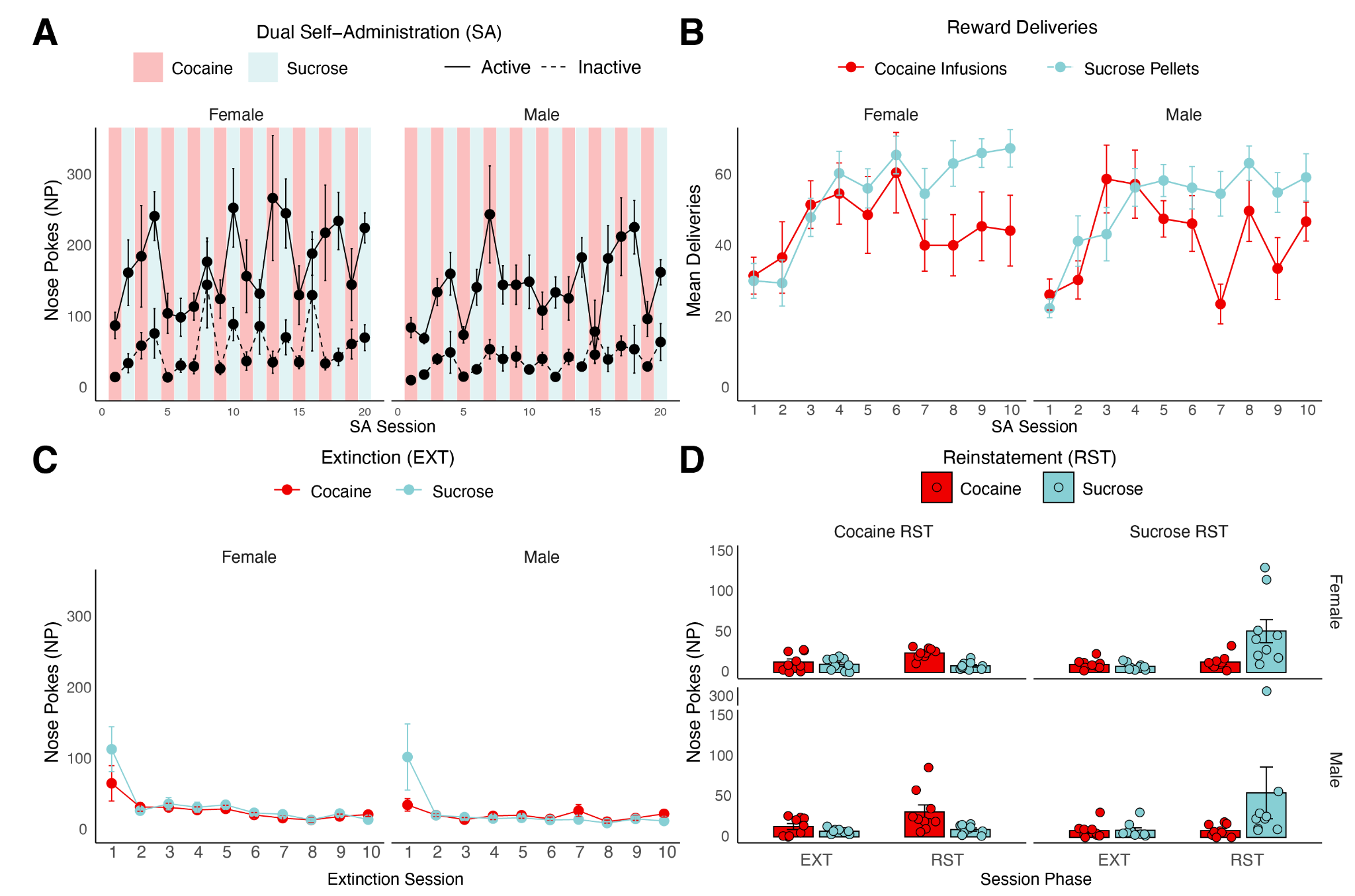


**Supplementary Figure 2. Sex-separated cocaine and sucrose self-administration, extinction, and cue-induced reinstatement behavior. A** Active and inactive nose pokes (NP) across 20 sessions of self-administration (SA), shown separately for females and males. Red and blue shaded bars indicate alternating days of cocaine or sucrose access. **B** Rewards delivered per session for cocaine (infusions) and sucrose (pellets) across the first 10 SA sessions, stratified by sex. **C** Cocaine- or sucrose-trained NP during extinction (EXT) sessions over 10 days, shown for each sex. **D** Cue-induced reinstatement (RST): active nose pokes during the first 30 minutes of the previous extinction session (EXT) versus cocaine- (C-RST) or sucrose-reinstatement (S-RST) session, separated by sex. Data shown as individual points with group means ± SEM.


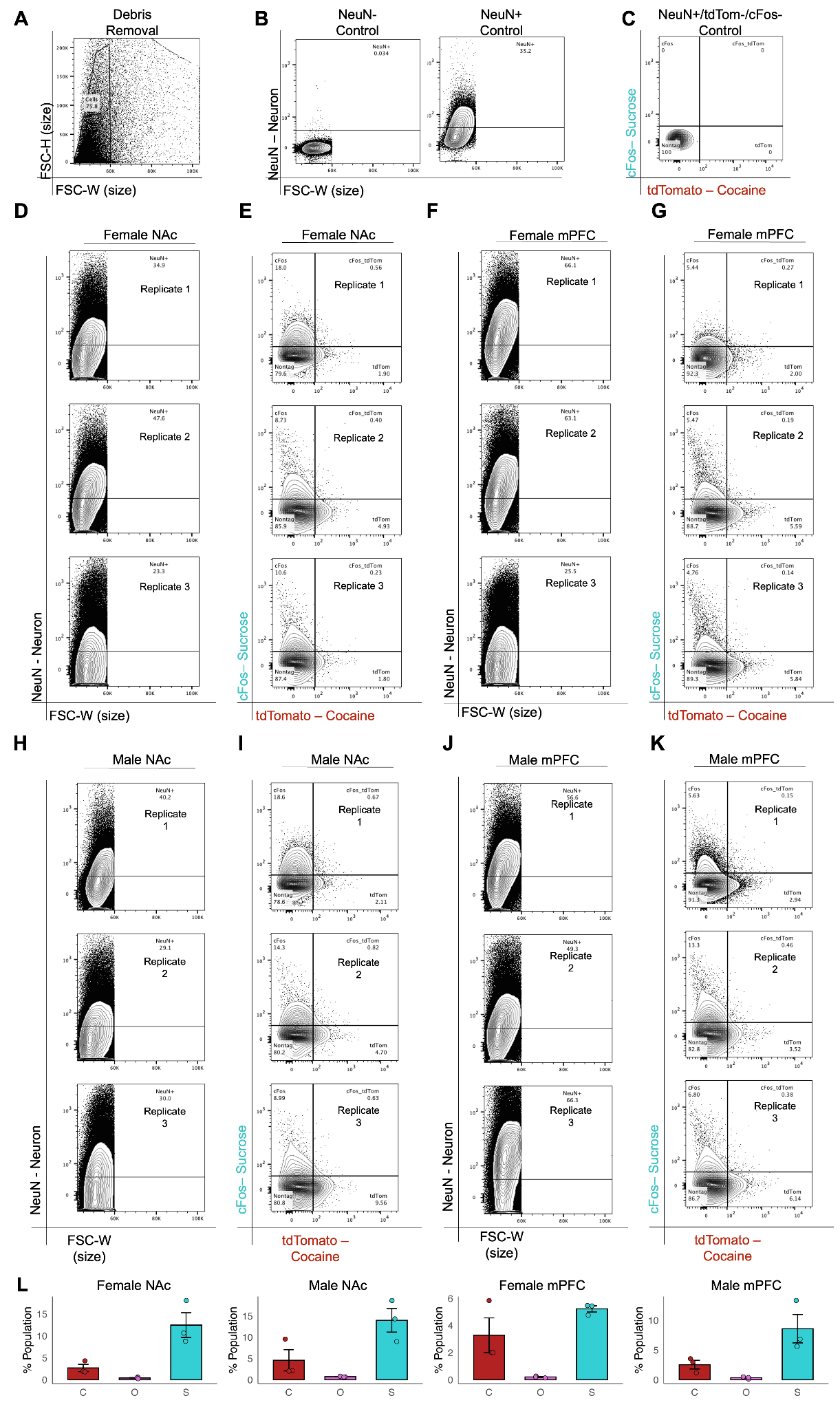


**Supplementary Figure 3. Fluorescence-activated cell sorting (FACS) strategy and ensemble gating for RNA-seq sample isolation. A** Size-based gating to remove debris using forward scatter height (FSC-H) and width (FSC-W). **B** Neuronal gating strategy using NeuN immunoreactivity, with NeuN⁺ (neuronal) and NeuN⁻ (non-neuronal) gates defined from control samples. **C** Control gating for ensemble-specific markers using NeuN⁺ cells from non-induced mice, establishing quadrant gates for tdTomato (cocaine-tagged; x-axis) and cFos (sucrose-tagged; y-axis) expression. **D–G** Female replicates: NeuN⁺ gating (**D,F**) and ensemble-specific quadrant separation in NAc (**E**) and mPFC (**G**). **H–K** Male replicates: NeuN⁺ gating (**H,J**) and ensemble-specific quadrant separation in NAc (**I**) and mPFC (**K**). Quadrants correspond to: non-ensemble (bottom left), cocaine-seeking ensemble (bottom right), sucrose-seeking ensemble (top left), and overlap ensemble (top right). **L** Quantification of FACS-identified populations across regions and sex. Bars represent mean ± SEM of percent total NeuN⁺ events per ensemble group.


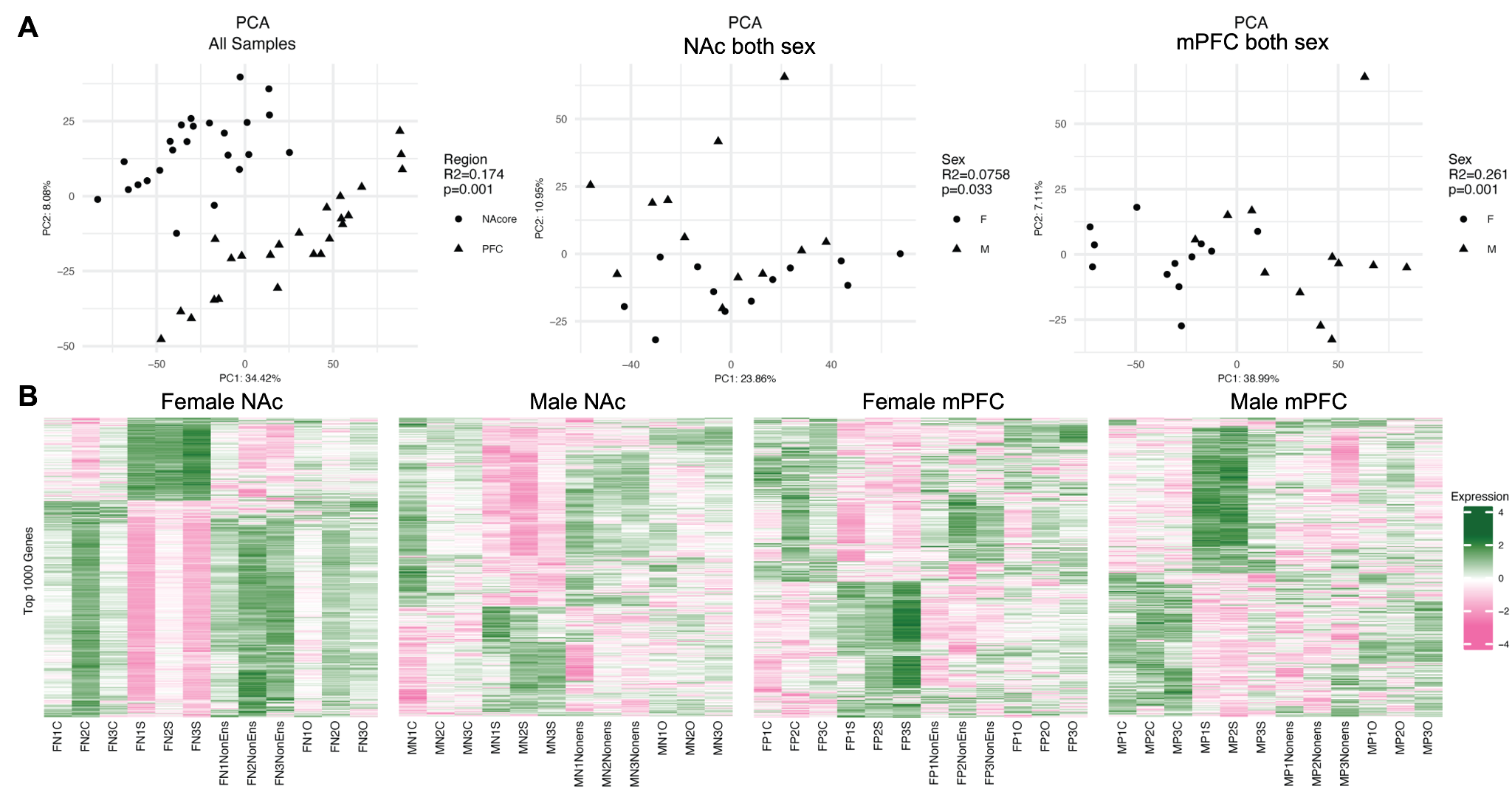


**Supplementary Figure 4. Transcriptomic dataset overview across brain region, sex, and reward condition.** **A** Principal component analysis (PCA) plots of log2-transformed CPM values grouped by region and sex. Statistical comparisons were performed using a permutative ANOVA to assess group-level separation. **B** Heatmaps showing z-scored (by row) expression of the top 1000 most variable genes across all samples. Columns represent pooled RNA-seq samples from three mice each, stratified by brain region (NAc or mPFC), sex (female or male), and reward condition (C = Cocaine, N = Non-Ensemble, O = Overlap, S = Sucrose).


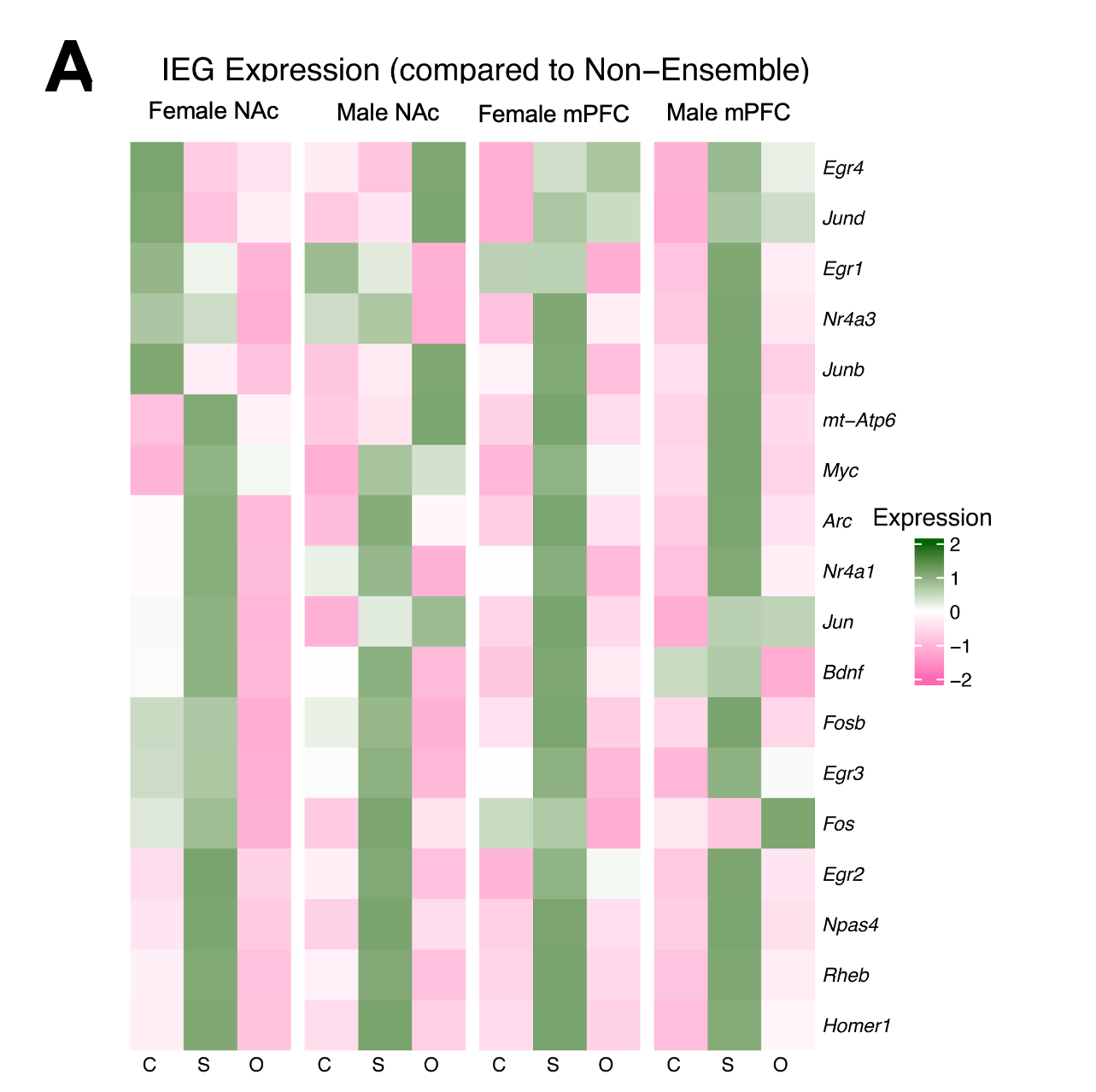


**Supplementary Figure 5. Differential expression of immediate early genes (IEGs) across reward ensembles and brain regions. A** Heatmap shows z-scored expression of IEGs relative to the non-ensemble group (baseline) for each reward condition: Cocaine (C), Sucrose (S), and Overlap (O). Expression values are stratified by brain region (NAc, mPFC) and sex (F, M). Rows represent selected genes involved in activity-dependent signaling and plasticity; columns represent ensemble type within each group. Gene expression was normalized per gene across conditions to highlight relative changes in expression compared to non-ensemble.
