# Supplementary material for: Corticostriatal cocaine-seeking ensembles are defined by differing gene expression from sucrose-seeking ensembles using a within-subject dual self-administration and seeking mouse model
Source: Addict Neurosci. Author manuscript; Available in PMC 2026 Jun 26. (PMC13298087; doi:10.1016/j.addicn.2025.100242)
Supplement: 3 [file NIHMS2186364-supplement-3.docx]

**Table 1A: Self-Administration Nose Pokes**

| Model | Description |
| --- | --- |
| Self-Administration (Active/Inactive Nose Poke) × Reward × Day | GLMM with full interactions and subject as random effect; Post-hoc Multiple Pairwise Comparisons |

**Fixed Effects**

| Term | Estimate | SE | z | p-value | Sig |
| --- | --- | --- | --- | --- | --- |
| Intercept | 3.182 | 0.207 | 15.36 | < 0.0001 | **** |
| Nose Poke (Active vs. Inactive) | 1.574 | 0.258 | 6.11 | < 0.0001 | **** |
| Reward (Sucrose vs. Cocaine) | 0.865 | 0.273 | 3.16 | 0.0016 | ** |
| Session (Day) | 0.030 | 0.014 | 2.12 | 0.0344 | * |
| Sex (Male vs Female) | 0.013 | 0.302 | 0.04 | 0.9656 | ns |
| Nose Poke × Reward | -0.523 | 0.354 | -1.48 | 0.1391 | ns |
| Nose Poke × Session | -0.010 | 0.021 | -0.50 | 0.6187 | ns |
| Reward × Session | -0.021 | 0.021 | -1.00 | 0.3188 | ns |
| Nose Poke × Sex | 0.050 | 0.373 | 0.13 | 0.8942 | ns |
| Reward × Sex | -0.902 | 0.386 | -2.34 | 0.0194 | * |
| Session × Sex | 0.021 | 0.022 | 0.98 | 0.3284 | ns |
| Nose Poke × Reward × Session | 0.017 | 0.030 | 0.59 | 0.5565 | ns |
| Nose Poke × Reward × Sex | 0.422 | 0.503 | 0.84 | 0.4011 | ns |
| Nose Poke × Session × Sex | -0.037 | 0.030 | -1.24 | 0.2168 | ns |
| Reward × Session × Sex | -0.001 | 0.030 | -0.04 | 0.9648 | ns |
| Nose Poke × Reward × Session × Sex | 0.033 | 0.042 | 0.78 | 0.4325 | ns |

**Random Effects**

| Group | Variance | SD |
| --- | --- | --- |
| Mouse | 0.0511 | 0.2260531 |

**Post-hoc Multiple Pairwise Comparison: Nose Poke × Reward × Sex**

| Reward | Sex | Contrast | Estimate | SE | z | adj. p value | Sig |
| --- | --- | --- | --- | --- | --- | --- | --- |
| Cocaine | Female | Active - Inactive | -1.466 | 0.155 | -9.46 | < 0.0001 | **** |
| Sucrose | Female | Active - Inactive | -1.126 | 0.154 | -7.29 | < 0.0001 | **** |
| Cocaine | Male | Active - Inactive | -1.122 | 0.153 | -7.33 | < 0.0001 | **** |
| Sucrose | Male | Active - Inactive | -1.552 | 0.154 | -10.10 | < 0.0001 | **** |

**Table 1B: Self-Administration Reward Deliveries**

| **Model** | **Description** |
| --- | --- |
| Delivery (Infusion/Pellet) × Session × Sex | GLMM with full interactions and subject as random effect; Post-hoc Multiple Pairwise Comparisons |

**Fixed Effects**

| **Term** | **Estimate** | **SE** | **z** | **p-value** | **Sig** |
| --- | --- | --- | --- | --- | --- |
| Intercept | 3.738 | 0.133 | 28.03 | < 0.0001 | **** |
| Reward (Sucrose vs. Cocaine) | -0.226 | 0.174 | -1.30 | 0.1943 | ns |
| Session (Day) | 0.009 | 0.019 | 0.48 | 0.6341 | ns |
| Sex (Male vs. Female) | -0.069 | 0.188 | -0.37 | 0.7142 | ns |
| Reward × Session | 0.073 | 0.026 | 2.74 | 0.0061 | ** |
| Reward × Sex | 0.070 | 0.247 | 0.28 | 0.7771 | ns |
| Session × Sex | 0.001 | 0.027 | 0.03 | 0.9789 | ns |
| Reward × Session × Sex | -0.011 | 0.037 | -0.30 | 0.7637 | ns |

**Random Effects**

| **Group** | **Variance** | **SD** |
| --- | --- | --- |
| Mouse | 0.0223 | 0.1493318 |

**Post-hoc Multiple Pairwise Comparison: Reward × Sex × Session**

| **Session** | **Sex** | **Contrast** | **Estimate** | **SE** | **z** | **adj. p value** | **Sig** |
| --- | --- | --- | --- | --- | --- | --- | --- |
| 1 | Female | Cocaine - Sucrose | 0.154 | 0.153 | 1.01 | 0.3147 | ns |
| 1 | Male | Cocaine - Sucrose | 0.095 | 0.153 | 0.62 | 0.5349 | ns |
| 2 | Female | Cocaine - Sucrose | 0.081 | 0.133 | 0.61 | 0.5430 | ns |
| 2 | Male | Cocaine - Sucrose | 0.034 | 0.133 | 0.25 | 0.8009 | ns |
| 3 | Female | Cocaine - Sucrose | 0.008 | 0.116 | 0.07 | 0.9431 | ns |
| 3 | Male | Cocaine - Sucrose | -0.028 | 0.116 | -0.24 | 0.8113 | ns |
| 4 | Female | Cocaine - Sucrose | -0.064 | 0.103 | -0.62 | 0.5326 | ns |
| 4 | Male | Cocaine - Sucrose | -0.089 | 0.103 | -0.86 | 0.3884 | ns |
| 5 | Female | Cocaine - Sucrose | -0.137 | 0.096 | -1.43 | 0.1533 | ns |
| 5 | Male | Cocaine - Sucrose | -0.151 | 0.096 | -1.57 | 0.1174 | ns |
| 6 | Female | Cocaine - Sucrose | -0.210 | 0.096 | -2.19 | 0.0286 | * |
| 6 | Male | Cocaine - Sucrose | -0.212 | 0.096 | -2.21 | 0.0273 | * |
| 7 | Female | Cocaine - Sucrose | -0.282 | 0.103 | -2.75 | 0.0060 | ** |
| 7 | Male | Cocaine - Sucrose | -0.273 | 0.103 | -2.65 | 0.0079 | ** |
| 8 | Female | Cocaine - Sucrose | -0.355 | 0.115 | -3.07 | 0.0021 | ** |
| 8 | Male | Cocaine - Sucrose | -0.335 | 0.116 | -2.89 | 0.0038 | ** |
| 9 | Female | Cocaine - Sucrose | -0.428 | 0.132 | -3.23 | 0.0012 | ** |
| 9 | Male | Cocaine - Sucrose | -0.396 | 0.133 | -2.99 | 0.0028 | ** |
| 10 | Female | Cocaine - Sucrose | -0.500 | 0.152 | -3.29 | 0.0010 | *** |
| 10 | Male | Cocaine - Sucrose | -0.457 | 0.152 | -3.00 | 0.0027 | ** |

**Table 1C: Extinction Nose Pokes**

| **Model** | **Description** |
| --- | --- |
| Extinction (Cocaine/Sucrose Nose Poke) × Session × Sex | GLMM with full interactions and subject as random effect; Post-hoc Multiple Pairwise Comparisons |

**Fixed Effects**

| **Term** | **Estimate** | **SE** | **z** | **p-value** | **Sig** |
| --- | --- | --- | --- | --- | --- |
| Intercept | 3.630 | 0.220 | 16.51 | < 0.0001 | **** |
| Nose Poke (Sucrose vs. Cocaine) | 0.387 | 0.198 | 1.96 | 0.0505 | . |
| Session (Day) | -0.089 | 0.016 | -5.45 | < 0.0001 | **** |
| Sex (Male vs Female) | -0.611 | 0.312 | -1.96 | 0.0502 | . |
| Nose Poke × Session | -0.046 | 0.023 | -1.99 | 0.0464 | * |
| Nose Poke × Sex | 0.099 | 0.281 | 0.35 | 0.7244 | ns |
| Session × Sex | 0.052 | 0.023 | 2.23 | 0.0258 | * |
| Nose Poke × Session × Sex | -0.047 | 0.033 | -1.44 | 0.1495 | ns |

**Random Effects**

| **Group** | **Variance** | **SD** |
| --- | --- | --- |
| Mouse | 0.2576 | 0.5075431 |

**Post hoc Multiple Pairwise Comparison: Nose Poke × Sex × Session**

| **Session** | **Sex** | **Contrast** | **Estimate** | **SE** | **z** | **FDR-adjusted p-value** | **Sig** |
| --- | --- | --- | --- | --- | --- | --- | --- |
| 1 | Female | Sucrose - Cocaine Nose Poke | -0.341 | 0.184 | -1.86 | 0.0632 | . |
| 1 | Male | Sucrose - Cocaine Nose Poke | -0.393 | 0.185 | -2.12 | 0.0336 | * |
| 2 | Female | Sucrose - Cocaine Nose Poke | -0.295 | 0.171 | -1.72 | 0.0848 | . |
| 2 | Male | Sucrose - Cocaine Nose Poke | -0.300 | 0.172 | -1.74 | 0.0822 | . |
| 3 | Female | Sucrose - Cocaine Nose Poke | -0.249 | 0.161 | -1.55 | 0.1223 | ns |
| 3 | Male | Sucrose - Cocaine Nose Poke | -0.207 | 0.162 | -1.27 | 0.2032 | ns |
| 4 | Female | Sucrose - Cocaine Nose Poke | -0.203 | 0.154 | -1.32 | 0.1871 | ns |
| 4 | Male | Sucrose - Cocaine Nose Poke | -0.114 | 0.155 | -0.73 | 0.4644 | ns |
| 5 | Female | Sucrose - Cocaine Nose Poke | -0.157 | 0.150 | -1.05 | 0.2947 | ns |
| 5 | Male | Sucrose - Cocaine Nose Poke | -0.020 | 0.151 | -0.14 | 0.8926 | ns |
| 6 | Female | Sucrose - Cocaine Nose Poke | -0.112 | 0.150 | -0.74 | 0.4566 | ns |
| 6 | Male | Sucrose - Cocaine Nose Poke | 0.073 | 0.151 | 0.48 | 0.6300 | ns |
| 7 | Female | Sucrose - Cocaine Nose Poke | -0.066 | 0.153 | -0.43 | 0.6678 | ns |
| 7 | Male | Sucrose - Cocaine Nose Poke | 0.166 | 0.154 | 1.08 | 0.2817 | ns |
| 8 | Female | Sucrose - Cocaine Nose Poke | -0.020 | 0.159 | -0.12 | 0.9016 | ns |
| 8 | Male | Sucrose - Cocaine Nose Poke | 0.259 | 0.161 | 1.61 | 0.1068 | ns |
| 9 | Female | Sucrose - Cocaine Nose Poke | 0.026 | 0.169 | 0.16 | 0.8764 | ns |
| 9 | Male | Sucrose - Cocaine Nose Poke | 0.352 | 0.170 | 2.07 | 0.0384 | * |
| 10 | Female | Sucrose - Cocaine Nose Poke | 0.072 | 0.180 | 0.40 | 0.6893 | ns |
| 10 | Male | Sucrose - Cocaine Nose Poke | 0.445 | 0.182 | 2.45 | 0.0144 | * |

**Table 1D: Self-Administration vs. Extinction Phases (Active Nose Poke)**

| **Model** | **Description** |
| --- | --- |
| Active Nose Poke (SA/EXT) × Reward × Sex | GLMM with full interactions and subject as random effect; Post-hoc Multiple Pairwise Comparisons |

**Fixed Effects**

| **Term** | **Estimate** | **SE** | **z** | **p-value** | **Sig** |
| --- | --- | --- | --- | --- | --- |
| Intercept | 4.953 | 0.157 | 31.47 | < 0.0001 | **** |
| Active Nose Poke (SA vs. EXT) | -1.774 | 0.094 | -18.95 | < 0.0001 | **** |
| Reward (Sucrose vs. Cocaine) | 0.344 | 0.106 | 3.26 | 0.0011 | ** |
| Sex (Male vs. Female) | -0.059 | 0.223 | -0.27 | 0.7902 | ns |
| Phase × Reward | -0.196 | 0.132 | -1.49 | 0.1362 | ns |
| Phase × Sex | -0.222 | 0.134 | -1.66 | 0.0966 | . |
| Reward × Sex | -0.160 | 0.149 | -1.07 | 0.2827 | ns |
| Phase × Reward × Sex | 0.005 | 0.188 | 0.03 | 0.9774 | ns |

**Random Effects**

| **Group** | **Variance** | **SD** |
| --- | --- | --- |
| Mouse | 0.1727 | 0.4155719 |

**Post-hoc Multiple Pairwise Comparison: Phase (SA/EXT) × Reward × Sex**

| **Reward** | **Sex** | **Contrast** | **Estimate** | **SE** | **z** | **FDR-adjusted p-value** | **Sig** |
| --- | --- | --- | --- | --- | --- | --- | --- |
| Cocaine | Female | EXT - SA | 1.774 | 0.094 | 18.95 | < 0.0001 | **** |
| Sucrose | Female | EXT - SA | 1.970 | 0.094 | 21.06 | < 0.0001 | **** |
| Cocaine | Male | EXT - SA | 1.996 | 0.095 | 20.94 | < 0.0001 | **** |
| Sucrose | Male | EXT - SA | 2.187 | 0.095 | 23.02 | < 0.0001 | **** |

**Table 1E: Previous Day Extinction vs. Reinstatement (Nose Poke)**

| **Model** | **Description** |
| --- | --- |
| Phase (EXT/RST) × Nose Poke × Sex | GLMM with full interactions and subject as random effect; Post-hoc Multiple Pairwise Comparisons |

**Fixed Effects**

| **Term** | **Estimate** | **SE** | **z** | **p-value** | **Sig** |
| --- | --- | --- | --- | --- | --- |
| Intercept | 2.372 | 0.272 | 8.73 | <0.0001 | **** |
| RST vs EXT | 0.816 | 0.260 | 3.14 | 0.0017 | ** |
| Nose Poke: Sucrose vs Cocaine | -0.190 | 0.272 | -0.70 | 0.4848 | ns |
| Sex: Male vs Female | -0.002 | 0.385 | -0.01 | 0.9958 | ns |
| EXT × Nose Poke | -0.070 | 0.391 | -0.18 | 0.8580 | ns |
| C-RST × Nose Poke | -0.968 | 0.380 | -2.55 | 0.0108 | * |
| S-RST × Nose Poke | 1.486 | 0.369 | 4.03 | <0.0001 | **** |
| EXT × Sex | -0.478 | 0.477 | -1.00 | 0.3168 | ns |
| C-RST × Sex | 0.103 | 0.368 | 0.28 | 0.7786 | ns |
| S-RST × Sex | -0.573 | 0.473 | -1.21 | 0.2263 | ns |
| Nose Poke × Sex | -0.251 | 0.392 | -0.64 | 0.5208 | ns |
| EXT × Nose Poke × Sex | 0.625 | 0.565 | 1.10 | 0.2693 | ns |
| C-RST × Nose Poke × Sex | 0.212 | 0.541 | 0.39 | 0.6956 | ns |
| S-RST × Nose Poke × Sex | 0.509 | 0.539 | 0.95 | 0.3442 | ns |

**Random Effects**

| **Group** | **Variance** | **SD** |
| --- | --- | --- |
| Mouse | 0.1738380 | 0.4169388 |

**Post hoc Multiple Pairwise Comparison: Phase (EXT-C, RST-C, EXT-S, RST-S) × Nose Poke × Sex**

| **Sex** | **contrast** | **Estimate** | **SE** | **z** | **adj. p-value** | **Sig** |
| --- | --- | --- | --- | --- | --- | --- |
| Female | (EXT-C Cocaine) - (EXT-S Cocaine) | 0.139 | 0.332 | 0.42 | 0.7556 | ns |
| Female | (EXT-C Cocaine) - (C-RST Cocaine) | -0.816 | 0.260 | -3.14 | 0.0048 | ** |
| Female | (EXT-C Cocaine) - (S-RST Cocaine) | -0.100 | 0.328 | -0.30 | 0.8192 | ns |
| Female | (EXT-C Cocaine) - (EXT-C Sucrose) | 0.190 | 0.272 | 0.70 | 0.6464 | ns |
| Female | (EXT-C Cocaine) - (EXT-S Sucrose) | 0.399 | 0.338 | 1.18 | 0.3927 | ns |
| Female | (EXT-C Cocaine) - (C-RST Sucrose) | 0.341 | 0.277 | 1.23 | 0.3824 | ns |
| Female | (EXT-C Cocaine) - (S-RST Sucrose) | -1.396 | 0.317 | -4.40 | < 0.0001 | **** |
| Female | (EXT-S Cocaine) - (C-RST Cocaine) | -0.956 | 0.323 | -2.96 | 0.0079 | ** |
| Female | (EXT-S Cocaine) - (S-RST Cocaine) | -0.239 | 0.270 | -0.88 | 0.5346 | ns |
| Female | (EXT-S Cocaine) - (EXT-C Sucrose) | 0.050 | 0.335 | 0.15 | 0.8805 | ns |
| Female | (EXT-S Cocaine) - (EXT-S Sucrose) | 0.260 | 0.282 | 0.92 | 0.5346 | ns |
| Female | (EXT-S Cocaine) - (C-RST Sucrose) | 0.201 | 0.338 | 0.60 | 0.6709 | ns |
| Female | (EXT-S Cocaine) - (S-RST Sucrose) | -1.535 | 0.257 | -5.97 | < 0.0001 | **** |
| Female | (C-RST Cocaine) - (S-RST Cocaine) | 0.717 | 0.319 | 2.25 | 0.0574 | . |
| Female | (C-RST Cocaine) - (EXT-C Sucrose) | 1.006 | 0.264 | 3.81 | 0.0005 | *** |
| Female | (C-RST Cocaine) - (EXT-S Sucrose) | 1.215 | 0.329 | 3.69 | 0.0007 | *** |
| Female | (C-RST Cocaine) - (C-RST Sucrose) | 1.157 | 0.267 | 4.34 | < 0.0001 | **** |
| Female | (C-RST Cocaine) - (S-RST Sucrose) | -0.580 | 0.308 | -1.88 | 0.1292 | ns |
| Female | (S-RST Cocaine) - (EXT-C Sucrose) | 0.289 | 0.331 | 0.87 | 0.5346 | ns |
| Female | (S-RST Cocaine) - (EXT-S Sucrose) | 0.499 | 0.279 | 1.79 | 0.1482 | ns |
| Female | (S-RST Cocaine) - (C-RST Sucrose) | 0.441 | 0.334 | 1.32 | 0.3498 | ns |
| Female | (S-RST Cocaine) - (S-RST Sucrose) | -1.296 | 0.250 | -5.18 | < 0.0001 | **** |
| Female | (EXT-C Sucrose) - (EXT-S Sucrose) | 0.209 | 0.341 | 0.61 | 0.6709 | ns |
| Female | (EXT-C Sucrose) - (C-RST Sucrose) | 0.151 | 0.279 | 0.54 | 0.6861 | ns |
| Female | (EXT-C Sucrose) - (S-RST Sucrose) | -1.586 | 0.321 | -4.94 | < 0.0001 | **** |
| Female | (EXT-S Sucrose) - (C-RST Sucrose) | -0.058 | 0.344 | -0.17 | 0.8805 | ns |
| Female | (EXT-S Sucrose) - (S-RST Sucrose) | -1.795 | 0.266 | -6.76 | < 0.0001 | **** |
| Female | (C-RST Sucrose) - (S-RST Sucrose) | -1.737 | 0.324 | -5.36 | < 0.0001 | **** |
| Male | (EXT-C Cocaine) - (EXT-S Cocaine) | 0.617 | 0.343 | 1.80 | 0.1548 | ns |
| Male | (EXT-C Cocaine) - (C-RST Cocaine) | -0.920 | 0.261 | -3.53 | 0.0011 | ** |
| Male | (EXT-C Cocaine) - (S-RST Cocaine) | 0.473 | 0.341 | 1.38 | 0.2908 | ns |
| Male | (EXT-C Cocaine) - (EXT-C Sucrose) | 0.441 | 0.283 | 1.56 | 0.2368 | ns |
| Male | (EXT-C Cocaine) - (EXT-S Sucrose) | 0.504 | 0.342 | 1.47 | 0.2620 | ns |
| Male | (EXT-C Cocaine) - (C-RST Sucrose) | 0.277 | 0.277 | 1.00 | 0.5090 | ns |
| Male | (EXT-C Cocaine) - (S-RST Sucrose) | -1.081 | 0.322 | -3.36 | 0.0018 | ** |
| Male | (EXT-S Cocaine) - (C-RST Cocaine) | -1.537 | 0.334 | -4.61 | < 0.0001 | **** |
| Male | (EXT-S Cocaine) - (S-RST Cocaine) | -0.144 | 0.295 | -0.49 | 0.7286 | ns |
| Male | (EXT-S Cocaine) - (EXT-C Sucrose) | -0.176 | 0.353 | -0.50 | 0.7286 | ns |
| Male | (EXT-S Cocaine) - (EXT-S Sucrose) | -0.113 | 0.295 | -0.38 | 0.7847 | ns |
| Male | (EXT-S Cocaine) - (C-RST Sucrose) | -0.340 | 0.347 | -0.98 | 0.5090 | ns |
| Male | (EXT-S Cocaine) - (S-RST Sucrose) | -1.698 | 0.271 | -6.27 | < 0.0001 | **** |
| Male | (C-RST Cocaine) - (S-RST Cocaine) | 1.393 | 0.332 | 4.19 | < 0.0001 | **** |
| Male | (C-RST Cocaine) - (EXT-C Sucrose) | 1.361 | 0.274 | 4.97 | < 0.0001 | **** |
| Male | (C-RST Cocaine) - (EXT-S Sucrose) | 1.423 | 0.332 | 4.29 | < 0.0001 | **** |
| Male | (C-RST Cocaine) - (C-RST Sucrose) | 1.197 | 0.263 | 4.56 | < 0.0001 | **** |
| Male | (C-RST Cocaine) - (S-RST Sucrose) | -0.161 | 0.311 | -0.52 | 0.7286 | ns |
| Male | (S-RST Cocaine) - (EXT-C Sucrose) | -0.032 | 0.351 | -0.09 | 0.9284 | ns |
| Male | (S-RST Cocaine) - (EXT-S Sucrose) | 0.031 | 0.295 | 0.10 | 0.9284 | ns |
| Male | (S-RST Cocaine) - (C-RST Sucrose) | -0.195 | 0.346 | -0.57 | 0.7286 | ns |
| Male | (S-RST Cocaine) - (S-RST Sucrose) | -1.554 | 0.273 | -5.69 | < 0.0001 | **** |
| Male | (EXT-C Sucrose) - (EXT-S Sucrose) | 0.062 | 0.351 | 0.18 | 0.9248 | ns |
| Male | (EXT-C Sucrose) - (C-RST Sucrose) | -0.164 | 0.288 | -0.57 | 0.7286 | ns |
| Male | (EXT-C Sucrose) - (S-RST Sucrose) | -1.523 | 0.331 | -4.59 | < 0.0001 | **** |
| Male | (EXT-S Sucrose) - (C-RST Sucrose) | -0.226 | 0.345 | -0.66 | 0.7286 | ns |
| Male | (EXT-S Sucrose) - (S-RST Sucrose) | -1.585 | 0.270 | -5.87 | < 0.0001 | **** |
| Male | (C-RST Sucrose) - (S-RST Sucrose) | -1.359 | 0.325 | -4.18 | < 0.0001 | **** |

**Table 1F: Fluorescent-Activated Cell Sorting (FACS)**

| **Model** | **Description** |
| --- | --- |
| Ensemble Size (Condition) × Region × Sex | Three-way ANOVA with full interactions; Post-hoc Multiple Pairwise Comparisons |

**Fixed Effects**

| **Effect** | **Sum Sq** | **Mean Sq** | **F value** | **p-value** | **Sig** |
| --- | --- | --- | --- | --- | --- |
| Condition | 588.96 | 294.48 | 38.50 | <0.0001 | **** |
| Region | 53.29 | 53.29 | 6.97 | 0.0144 | * |
| Sex | 10.39 | 10.39 | 1.36 | 0.2553 | ns |
| Condition × Region | 67.66 | 33.83 | 4.42 | 0.0232 | * |
| Condition × Sex | 8.53 | 4.27 | 0.56 | 0.5798 | ns |
| Region × Sex | 0.24 | 0.24 | 0.03 | 0.8618 | ns |
| Condition × Region × Sex | 7.48 | 3.74 | 0.49 | 0.6192 | ns |
| Residuals | 183.59 | 7.65 |  |  | ns |

**Post hoc pairwise comparisons: Condition × Region**

| **Region** | **contrast** | **estimate** | **SE** | **t** | **adj. p-value** | **Sig** |
| --- | --- | --- | --- | --- | --- | --- |
| NAc | C - O | 3.07 | 1.6 | 1.92 | 0.1550 | ns |
| NAc | C - S | -9.59 | 1.6 | -6.00 | <0.0001 | **** |
| NAc | O - S | -12.65 | 1.6 | -7.92 | <0.0001 | **** |
| mPFC | C - O | 2.64 | 1.6 | 1.65 | 0.2431 | ns |
| mPFC | C - S | -3.99 | 1.6 | -2.50 | 0.0496 | * |
| mPFC | O - S | -6.63 | 1.6 | -4.16 | 0.0010 | ** |

**Table 1G: Reinstatement (Nose Poke) of Pooled Samples**

| **Model** | **Description** |
| --- | --- |
| Nose Poke x Phase (C-RST; S-RST) Sex x Replicate | GLMM Likelihood Ratio Test for replicate as random effect |

**Random Effects**

| **Effect** | **Χ²** | **p-value** |
| --- | --- | --- |
| Replicate | 1.53 | 0.4652 |
